# Network meta-analysis of multiple outcome measures accounting for borrowing of information across outcomes

**DOI:** 10.1186/1471-2288-14-92

**Published:** 2014-07-21

**Authors:** Felix A Achana, Nicola J Cooper, Sylwia Bujkiewicz, Stephanie J Hubbard, Denise Kendrick, David R Jones, Alex J Sutton

**Affiliations:** 1 Biostatistics Group, Department of Health Sciences, University of Leicester, University Road, Leicester LE1 7RH, UK; 2 Division of Primary Care Community Health Sciences, Faculty of Medicine & Health Sciences, University of Nottingham, Nottingham NG7 2RD, UK

**Keywords:** Network meta-analysis, Mixed treatment comparisons, Multiple outcomes, Multivariate, WinBUGS

## Abstract

**Background:**

Network meta-analysis (NMA) enables simultaneous comparison of multiple treatments while preserving randomisation. When summarising evidence to inform an economic evaluation, it is important that the analysis accurately reflects the dependency structure within the data, as correlations between outcomes may have implication for estimating the net benefit associated with treatment. A multivariate NMA offers a framework for evaluating multiple treatments across multiple outcome measures while accounting for the correlation structure between outcomes.

**Methods:**

The standard NMA model is extended to multiple outcome settings in two stages. In the first stage, information is borrowed across outcomes as well across studies through modelling the within-study and between-study correlation structure. In the second stage, we make use of the additional assumption that intervention effects are exchangeable between outcomes to predict effect estimates for all outcomes, including effect estimates on outcomes where evidence is either sparse or the treatment had not been considered by any one of the studies included in the analysis. We apply the methods to binary outcome data from a systematic review evaluating the effectiveness of nine home safety interventions on uptake of three poisoning prevention practices (safe storage of medicines, safe storage of other household products, and possession of poison centre control telephone number) in households with children. Analyses are conducted in WinBUGS using Markov Chain Monte Carlo (MCMC) simulations.

**Results:**

Univariate and the first stage multivariate models produced broadly similar point estimates of intervention effects but the uncertainty around the multivariate estimates varied depending on the prior distribution specified for the between-study covariance structure. The second stage multivariate analyses produced more precise effect estimates while enabling intervention effects to be predicted for all outcomes, including intervention effects on outcomes not directly considered by the studies included in the analysis.

**Conclusions:**

Accounting for the dependency between outcomes in a multivariate meta-analysis may or may not improve the precision of effect estimates from a network meta-analysis compared to analysing each outcome separately.

## Background

Meta-analysis or the quantitative synthesis of evidence, usually from systematic reviews, has become a popular tool in healthcare evaluations [1,2]. Largely driven by a desire for more realistic synthesis of complex healthcare evidence, increasingly sophisticated methodology has been developed. One area of meta-analysis that has seen significant methodological development is the application of multivariate statistical methods for the comparison of treatments on two or more endpoints (usually known as multivariate meta-analysis) [3-8]. These methods are appealing because many studies and systematic reviews focus on broad health effects and therefore typically report several outcome measures [4,6,9]. In such instances, the multivariate approach offers some advantages over separate univariate analyses including the ability to account for the inter-relationship between outcomes and borrow strength across studies as well as across outcomes [10] through modelling the correlation structure [7,11]. This can potentially reduce outcome reporting bias [12] and the uncertainty with which intervention effects are estimated. Additionally, in a decision making context where the synthesis is meant to inform a health economic evaluation, accounting for the correlations between effect estimates on different outcomes is important as the dependence between outcomes may have implication for estimating quality of life or economic consequences associated with treatment [13]. An example is the situation where a particularly effective treatment for a disease condition is associated with a large side effect profile. Ignoring information about the inter-relationships between beneficial and ‘side effect’ endpoints in such instances may have implications for quantifying the benefits associated with treatment.

When summarising effectiveness evidence, correlations between the effectiveness estimates typically arise at either within-study and or between-study levels. At the within-study level, correlations arise mainly due to differences in patient-level characteristics. They are rarely reported in the published literature and usually have to be estimated from external sources such as individual patient level data if available or elicited from expert opinion [8,11,14,15]. At the between-study level, correlations arise from i) differences in the distribution of patient-level characteristics across studies, in which case they will be related to the within-study correlations and/or ii) differences in the distribution of other study-level characteristics such as study design, population and baseline disease severity [16]. The within-study correlations thus give an indication of the association between multiple endpoints within a study while the between-study correlations indicate how the underlying true study-specific effects on different outcomes vary jointly across studies.

A second area of rapid methodological development is network meta-analysis (NMA) [17], also known as mixed treatment comparison meta-analysis [18-20] or multiple treatment meta-analysis [21-23]. NMA methods extend standard pairwise meta-analysis to enable simultaneous comparison of multiple treatments while maintaining randomisation of individual studies [18]. The method enables ‘direct’ evidence (i.e. evidence from studies directly comparing two interventions of interest) and ‘indirect’ evidence (i.e. evidence from studies that do not compare the two interventions directly) to be pooled under the assumption of evidence consistency [24]. Estimates of intervention effects can then be obtained, including effects between treatments not directly compared within any one individual study [19]. NMA methods thus provide a coherent framework for appraising all available evidence relevant to a specific decision problem. The results from such analyses are increasingly being used to inform economic evaluations in healthcare decision making where coherent decisions (about judicious use of scarce resource) need to be made based on sound appraisal of all available evidence.

Approaches to extend NMA methodology to multiple outcome settings have been proposed in the literature [13,25-27], initially focusing on mutually exclusive competing risk outcomes [13] or a single outcome measured at multiple time points [26,28]. More recently, Efthimiou *et al.*[14] proposed a method for modelling multiple correlated outcomes in networks of evidence with binary outcome measures. The proposed method accounts for both the within-study and between-study correlation structure and includes a strategy for eliciting expert opinion to inform the within-study correlations. This paper contributes to the growing literature on the simultaneous evaluation of correlated outcomes. We do this in two stages. In the first stage (labelled as model 2 in the remainder of the paper), information is borrowed across studies as well as across outcomes through modelling the correlations between effectiveness estimates on different outcomes. In the second stage (labelled as model 3 in the remainder of the paper), additional information is borrowed across outcomes based on ideas for combining evidence across human and animal studies originally proposed by DuMouchel and Harris [29] and also revisited by Jones *et al.*[30]. The proposed second stage analysis methods allows: i) disconnected treatments to be incorporated as nodes in a network of evidence and ii) prediction of intervention effects for outcomes where evidence from primary studies is either sparse or not directly available from any one study included in the analysis. The motivating application area is injury prevention in children where a broad array of outcomes and intervention packages have been evaluated with the aim of increasing safety practices around the home (to ultimately reduce household injuries).

The remainder of this paper is structured as follows: the example dataset is first described followed by a Methods section describing the statistical models developed and implementation of the models. These are followed by sections presenting the results of applying the methods to the motivating dataset and a discussion.

### Dataset

The example data comes from a recently updated Cochrane systematic review of home safety education and provision of safety equipment for injury prevention in children [31]. The models developed in this paper are applied to a subset of the review evidence relating to the prevention of poisoning injuries. Table 1 presents the data from 22 studies for the following outcomes:

a) Safe storage of medicines

b) Safe storage of other household products (e.g. cleaning products) and

c) Possession of a poison control centre (PCC) telephone number.

**Table 1:** Summary of the available evidence

			**Outcome information (no. of events/no. of households in control versus (vs.) treatment arm)**		
Comparison	First author and year of publication	IPD	Safe storage of medicines	Safe storage of other household products	Possession of a PCC number
Usual care (1) *vs.* Education (2)	Gielen 2007	Yes	178/271 vs. 188/249^Ɨ^	44/62 vs. 57/73^Ɨ^	
	Nansel 2002	Yes	83/89 vs. 79/85	65/89 vs. 66/85	59/89 vs. 63/85
	Nansel 2008	Yes	72/74 vs. 140/144^†^	59/73 vs. 117/144^†^	50/59 vs. 90/119^†^
	Kelly B 1987	No	54/54 vs. 55/55	43/54 vs. 49/55	
	McDonald 2005	No	4/57 vs. 6/60	3/57 vs. 6/61	
	Kelly N 2003	No			45.56/136.68 vs. 112.95/137.63^*^
Usual care (1) *vs*. Education + free/low cost safety equipment (3)	Clamp 1998	Yes	68/82 vs. 79/83	49/82 vs. 59/83	
	Woolf 1987	No			29/143 vs. 47/119
	Woolf 1992	No		60/151 vs. 89/150	59/151 vs. 117/150
Usual care (1) *vs.* Education + equipment (3) *vs*. Education + equipment + home safety inspection (4)	Babul 2007	Yes	147/149 vs. 171/173 vs. 160/163		
Usual care (1) *vs.* Education + equipment + home safety inspection (4)	Hendrickson 2002	Yes		14/40 vs. 34/38	8/40 vs. 34/38
	Swart 2008	No	70.26/79.58 vs. 74.07/80^*^	46.86/57.96 vs. 50.87/58.27^*^	
	Kendrick 1999	Yes		317/367 vs. 322/363	
Usual care (1) *vs.* Education + equipment + fitting (5)	Watson 2005	Yes	683/738 vs. 712/762	327/669 vs. 368/693	
Usual care (1) *vs.* Education + home safety inspection (6)	Petridou 1997	No			67.26/100.12 vs. 71.08/97.83^*^
Usual care (1) *vs.* Education + equipment + home safety inspection + fitting (7)	Schwarz D 1993	No	88.42/248.37 vs. 128.16/248.37^*^		
	Phelan 2011	No			16/138 vs. 71/139
Usual care (1) vs*.* Home visit (8)	Johnson 2006	No			82/91 vs. 222/232^†^
Education (2) *vs.* Education + equipment (3)	Posner 2004	Yes	14/47 vs. 19/49	22/47 vs. 34/49	27/47 vs. 35/49
Education (2) vs. Education + equipment + fitting (5)	Sznajder 2003	Yes	44/49 vs. 43/45	32/41 vs. 40/48	
Education + equipment + home safety inspection (4) vs. Education + equipment + home safety inspection + fitting (7)	King J 2001	No		261/469 vs. 273/482	
Education + equipment (3) vs. Equipment (9)	Dershewitz 1979	No	22/101 vs. 20/104	1/101 vs. 0/104	

Treatment abbreviation and codes:

Usual care = UC (1).

Education = E (2).

Education + free/low cost equipment = E + FE (3).

Education + equipment + home safety inspection = E + FE + HSI (4).

Education + equipment + fitting = E + FE + F (5).

Education + home safety inspection = E + HSI (6).

Education + equipment + home safety inspection + fitting = E + FE + HSI + F (7).

Education + home visit = E + HV (8).

Free/low cost equipment = FE (9).

*Effective sample size reported for cluster randomised studies after adjusting clustering, hence not whole numbers (details given in Kendrick *et al.* 2012 [31]).

^Ɨ^The IPD for Gielen 2007 shows information on safe storage of medicines and safe storage of other household products was collected from different sets of households in this study (i.e. all the households that provided information for storage of medicines had missing data for safe storage of other household products and vice versa). Hence the Gielen 2007 IPD was not used to estimate the within-study correlations. ^†^The intervention arms of Nansel 2008 and Johnson 2006 [32] comprises two groups that received different versions of a home safety intervention. The two versions were considered to be similar, hence combined into one intervention group for the analysis reported here.

Thirteen of the 22 studies considered at least two of the three outcomes. Of these, 8 considered storage of medicines and storage of other household products, 2 considered storage of other household products and possession of a PCC telephone number, and 3 considered all three outcome measures. Individual patient data (IPD) were available for 8 of the 13 studies, of which 7 were in a format suitable for the analysis reported here as explained by the footnotes in Table 1.

We classified the interventions trialled in the 22 studies into 9 relatively homogenous treatment packages:

1) Usual care (UC)

2) Education (E)

3) Education + provision of free/low cost equipment (E + FE)

4) Education + provision of free/low cost equipment + home safety inspection (E + FE + HSI)

5) Education + provision of free/low cost equipment + fitting of equipment (E + FE + F)

6) Education + home safety inspection (E + HSI)

7) Education + provision of free/low cost equipment + home safety inspection + fitting of equipment (E + FE + HSI + F)

8) Education + home visit (E + HV)

9) Provision of free/low cost equipment (FE).

**Figure 1:**
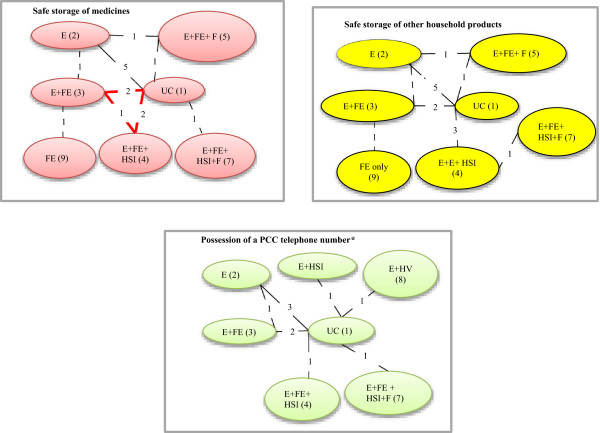
**Intervention networks for the poisoning prevention outcomes (thick red lines indicate multi-arm comparisons).** *PCC= poison control centre telephone number.

Figure 1 shows the comparisons between the interventions that were made by individual studies and the number of comparisons in each network. All studies compared 2 intervention strategies, except Babul *et al.* (2007) [33] which compared 3 strategies. Data on each outcome was not available for all interventions; i.e. for the storage of medicines and other household products outcomes, interventions E + HSI and E + HV were not investigated in any of the included studies, and for possession of a PCC number interventions, E + FE + F and FE were not available.

## Methods

In this section, we first present the NMA statistical model for one binary outcome measure and then extend it to compare multiple interventions across multiple outcomes. Throughout the paper, we refer to these single and multiple outcome models as univariate and multivariate NMAs respectively. Where studies report multiple outcomes, these will not be independent as each household provides information on the different outcome measures within intervention arms. The multivariate model takes this correlation structure into account by allowing the intervention effects measured by one outcome to be correlated with the intervention effects measured by other outcomes.

### Model 1: Univariate NMA

Given arm-level binary data of the form presented in Table 1, a random effects NMA may be specified using the method of Lu and Ades [20]. It is assumed that the occurrence of *r*_*ik*_ events out of a total of *n*_*ik*_ households in the *k*th-arm (*k = A,B,C,….,*) of the *i*th-study follow a binomial distribution with underlying event probability *p*_*ik*_:

(1) $$\begin{array}{l} r_{\mathrm{ik}}\;\sim \;\mathrm{Binomial}\left(p_{\mathrm{ik}},\;n_{\mathrm{ik}}\right);\;\mathrm{logit}\left(p_{\mathrm{ik}}\right)\;=\theta_{\mathrm{ik}}\\ \begin{array}{rl} \theta_{\mathrm{ik}} & =\left\{\begin{array}{c} \mu_{\mathrm{ib}}\;\\ \mu_{\mathrm{ib}}+\delta_{i\left(\mathrm{bk}\right)} \end{array}\right)\;\begin{array}{l} \mathrm{if}\;k=b\\ \mathrm{if}\;k>\;b \end{array}\\ b & =A,B,C, \end{array} \end{array}$$

(2) $$\begin{array}{lcl} \delta_{i\left(\mathrm{bk}\right)} & \sim & \mathrm{Normal}\left(d_{\left(\mathrm{bk}\right)}=d_{\left(\mathrm{Ak}\right)}-d_{\left(\mathrm{Ab}\right)}\;,\;\sigma_{\left(\mathrm{bk}\right)}^{2}\right)\\ \mathrm{Note} & : & d_{\mathrm{AA}}=0 \end{array}$$

where *μ*_*ib*_ is a study-specific baseline effect (i.e. the log-odds for the control group in study *i* with baseline treatment *b*), _*i(bk)*_ is a study-specific log-odds ratio, *d*_*(bk)*_ is the pooled effect of treatment *k* relative to treatment *b* (a quantity usually of interest in a meta-analysis) and $\sigma_{\left(\mathrm{bk}\right)}^{2}$ is the between-study variance or heterogeneity parameter. Random effects NMA assumes that intervention effects are exchangeable across the network regardless of whether or not treatments *b* and *k* are included in study *i*[18]. This assumption implies that the pooled effects *d*_*(bk)*_, can be expressed as functions of basic parameters with reference to a common comparator or baseline treatment (i.e. *d*_(*bk*)_ = *d*_(*Ak*)_ − *d*_(*Ab*)_) [24]. Throughout this paper, we take usual care (UC) intervention to be the reference or ‘baseline’ treatment (i.e. UC is taken as treatment *A* of equation (2) above). Multi-arm studies (i.e. studies with more than 2 treatment groups) present a special problem in network meta-analysis because they produce evidence on multiple treatment effects that are correlated through sharing a common reference or ‘baseline’ treatment. Under a homogenous variance assumption ($\sigma_{\left(\mathrm{bk}\right)}^{2}=\sigma^{2}$), the covariance between any two effects that share a common reference treatment is $\frac{\sigma^{2}}{2}$[20]. The homogeneous variance assumption allows for the distribution of effects (in a study with an arbitrary number of arms) to be expressed as a univariate marginal distribution and a series of univariate conditional distributions. Specifically, for the *i*th-study with *p + 1* arms and *p* treatment effect estimates relative to the reference treatment, if

(3) $$\begin{array}{l} \left(\begin{array}{c} \begin{array}{c} \begin{array}{c} \delta_{i\left(bk_{1}\right)}\\ \delta_{i\left(bk_{2}\right)} \end{array}\\ \vdots \end{array}\\ \delta_{i\left(bk_{p}\right)} \end{array}\right)\sim \mathrm{Normal}\left(\left(\begin{array}{c} \begin{array}{c} \begin{array}{c} d_{\left(bk_{1}\right)}\\ d_{\left(bk_{2}\right)} \end{array}\\ \vdots \end{array}\\ d_{\left(bk_{p}\right)} \end{array}\right),\right)\\ \left(\left(\begin{array}{c} \begin{array}{c} \sigma^{2}\\ \frac{\sigma^{2}}{2} \end{array}\\ \begin{array}{c} \vdots \\ \frac{\sigma^{2}}{2} \end{array} \end{array}\begin{array}{c} \begin{array}{c} \frac{\sigma^{2}}{2}\\ \sigma^{2} \end{array}\\ \begin{array}{c} \vdots \\ \frac{\sigma^{2}}{2} \end{array} \end{array}\begin{array}{c} \begin{array}{c} \cdots \\ \cdots \end{array}\\ \begin{array}{c} \ddots \\ \cdots \end{array} \end{array}\begin{array}{c} \begin{array}{c} \frac{\sigma^{2}}{2}\\ \frac{\sigma^{2}}{2} \end{array}\\ \begin{array}{c} \vdots \\ \sigma^{2} \end{array} \end{array}\right)\right) \end{array}$$

then the marginal and conditional univariate distributions for arm *j*, given the previous 1, ⋯, (*j* − 1) arms are:

(4) $$\begin{array}{l} \delta_{i\left(bk_{1}\right)}\sim \mathrm{Normal}\left(d_{\left(bk_{1}\right)}\;,\;\sigma^{2}\right)\;\mathrm{for}\;j=1.\\ \delta_{i\left(bk_{j}\right)}|\left(\begin{array}{c} \delta_{i\left(bk_{1}\right)}\\ \begin{array}{c} \vdots \\ \delta_{i\left(bk_{j-1}\right)} \end{array} \end{array}\right)\sim \mathrm{Normal}\left(d_{\left(bk_{j}\right)}+\frac{1}{j}\sum_{t=1}^{j-1}\left(\delta_{i\left(bk_{t}\right)}-d_{\left(bk_{t}\right)}\right),\;\frac{\left(j+1\right)}{2j}\sigma^{2}\right)\\ \;\mathrm{for}\;j=2,\ldots ,p \end{array}$$

The analysis is conducted within a Bayesian framework requiring prior distributions to be specified for all model parameters. Accordingly, we specified minimally informative prior distributions corresponding to a Normal (0,10^3^) prior distribution for the pooled mean effects relative to usual care, *d*_(*Ak*)_ and the study-specific baseline effects, *μ*_*ib*_ and a Uniform (0,2) prior distribution for the between-study standard deviation on the log odds ratio scale *σ*[34].

### Model 2: Multivariate NMA

We extend the univariate NMA model defined above to the multiple outcomes settings in order to account for correlations between intervention effects on different outcomes. The method presented here follows from Ades *et al.* (2010) NMA with competing risks model [13] where only the within-study correlations are taken into account. We extend their method to account for the between-study correlation as well.

Note that in Ades *et al.* (2010), a multinomial likelihood was appropriate as the three binary outcomes (relapse during treatment for Schizophrenia, discontinuation because of intolerable side effects, and discontinuation for other reasons) are mutually exclusive and event probabilities sum to 1 across outcomes. A multinomial likelihood will not be appropriate for our example dataset because each household can have one, two or all three outcomes simultaneously so that the event probabilities do not sum to 1 across outcomes. Instead, we use a Normal distribution on the log-odds scale to take account of the within-study correlations between outcomes. We assume that in each study *i* and for each *k*-th arm, the estimates ${\hat{\theta }}_{\mathrm{ikm}}$ of the observed log-odds of an event on the *m*th outcome (*m* = 1, 2, ⋯, *M*) jointly follow a multivariate normal distribution:

(5) $$\begin{array}{l} \begin{array}{rcl} \left(\begin{array}{c} \begin{array}{c} {\hat{\theta }}_{\mathrm{ik}1}\\ \vdots \end{array}\\ {\hat{\theta }}_{\mathrm{ikM}} \end{array}\right) & \sim & \mathrm{Normal}\left(\left(\begin{array}{c} \begin{array}{c} \theta_{\mathrm{ik}1}\\ \vdots \end{array}\\ \theta_{\mathrm{ikM}} \end{array}\right),\right)\\ S_{\mathrm{ik}} & = & \left(\left(\begin{array}{ccc} s_{\mathrm{ik}1}^{2} & \cdots \; & r_{\mathrm{ik}}^{1M}s_{\mathrm{ik}1}s_{\mathrm{ikM}}\;\\ & \ddots & \vdots \;\\ & & s_{\mathrm{ikM}}^{2} \end{array}\right)\right)\\ \left(\begin{array}{c} \begin{array}{c} \theta_{\mathrm{ik}1}\\ \vdots \end{array}\\ \theta_{\mathrm{ikM}} \end{array}\right) & = & \left\{\begin{array}{c} \left(\begin{array}{c} \begin{array}{c} \mu_{\mathrm{ib}1}\\ \vdots \end{array}\\ \mu_{\mathrm{ibM}} \end{array}\right)\;\\ \left(\begin{array}{c} \begin{array}{c} \mu_{\mathrm{ib}1}+\delta_{i\left(\mathrm{bk}\right)1}\\ \vdots \end{array}\\ \mu_{\mathrm{ibM}}+\delta_{i\left(\mathrm{bk}\right)M} \end{array}\right) \end{array}\right)\;\begin{array}{c} \mathrm{if}\;k=b\\ \mathrm{if}\;k>\;b\; \end{array} \end{array}\\ \mathrm{for}\;b=A,B,C,\;\ldots \end{array}$$

where (*μ*_*ib*1_, *μ*_*ib*2_, ⋯, *μ*_*ibM*_) and (*δ*_*i*(*bk*)1_, *δ*_*i*(*bk*)2_, ⋯, *δ*_*i*(*bk*)*M*_) represent vectors of *‘true’* baseline and study-specific effects in study *i* with baseline treatment *b* respectively. The quantities $\left({\hat{\theta }}_{\mathrm{ik}1},\;{\hat{\theta }}_{\mathrm{ik}2},\cdots ,\;\theta_{\mathrm{ikM}}\right)$ and (*θ*_*ik*1_, *θ*_*ik*2_, ⋯, *θ*_*ikM*_) represent vectors of observed and ‘*true’* log-odds of response in arm *k* of study *i* and **S**_*ik*_ is the associated within-study covariance matrix usually assumed known but estimated in practice from the data [35]. We calculated $\left({\hat{\theta }}_{\mathrm{ik}1},\;{\hat{\theta }}_{\mathrm{ik}2}\;,\cdots ,\;{\hat{\theta }}_{\mathrm{ikM}}\right)$ and the diagonal elements of **S**_*ik*_ using standard formulae for log-odds and variance of the log-odds [2]. We applied a continuity correction by adding 0.5 to the numerators and 1 to the denominators of studies with 0% or 100% event rate in one of the treatment arms [36,37]. The off-diagonals of **S**_*ik*_ were calculated from estimates of within-study correlations $r_{\mathrm{ik}}^{\mathrm{mn}}$ between outcomes *m* and *n* (*m ≠ n*) in arm *k* of study *i* obtained from studies with IPD (see Additional file 1: Table S1). The method used to estimate the correlations from the IPD is described in the implementation section below.

When summarising evidence across multiple endpoints, it is common to encounter instances where some studies do not report information for all outcomes of interest leading to incomplete vectors with missing study-specific effects for the outcomes not reported [5,10]. Such studies can be included in our model under the assumption that the effects for outcomes not reported are missing at random. When implemented using the WinBUGS software, the missing study effects and standard errors are coded as NA in the data, a strategy previously outlined in Bujkiewicz *et al.*[10] and Dakin *et al.*[28]. This enables WinBUGS to automatically ‘impute’ values for the missing information under missing at random assumption with predicted distributions.

We refer to equation (5) as the within-study model and the model describing the distribution of the *‘true’* effects across studies (presented below) as the between-study model following standard terminology in multivariate meta-analysis [5,6,10,11,38,39]. For the network of two-arm trials, the between-study model for the *i*th study is thus given by:

(6) $$\begin{array}{rcl} \left(\begin{array}{c} \begin{array}{c} \delta_{i\left(\mathrm{bk}\right)1}\\ \vdots \end{array}\\ \delta_{i\left(\mathrm{bk}\right)M} \end{array}\right) & \sim & \mathrm{Normal}\left(\left(\begin{array}{c} \begin{array}{c} d_{\left(\mathrm{bk}\right)1}=d_{\left(\mathrm{Ak}\right)1}-d_{\left(\mathrm{Ab}\right)1}\\ \vdots \end{array}\\ d_{\left(\mathrm{bk}\right)M}=d_{\left(\mathrm{Ak}\right)M}-d_{\left(\mathrm{Ab}\right)M} \end{array}\right),\;\Sigma_{\left(\mathrm{bk}\right)}\right)\\ \Sigma_{\left(\mathrm{bk}\right)} & = & \left(\begin{array}{ccc} \sigma_{\left(\mathrm{bk}\right)1}^{2} & \cdots \; & \rho_{\mathrm{bk}}^{1M}\sigma_{\left(\mathrm{bk}\right)1}\sigma_{\left(\mathrm{bk}\right)M}\;\\ & \ddots & \vdots \;\\ & & \sigma_{\left(\mathrm{bk}\right)M}^{2} \end{array}\right) \end{array}$$

where the *‘true’* effects *δ*_*i*(*bk*)*m*_ (*m* = 1, 2, ⋯, *M*) jointly follow a Normal distribution with mean effects *d*_(*bk*)*m*_. The parameters in equation (6) have the same interpretation as in equation (2) except that they are now specific to each outcome. The covariance matrix *Σ*_(*bk*)_ contains terms for the between-study variances, $\sigma_{\left(\mathrm{bk}\right)m}^{2}$ for each outcome *m* and the between-study correlations $\rho_{\mathrm{bk}}^{\mathrm{mn}}$ between effects measured by outcome *m* and *n* (*m ≠ n*) specific to each *k* versus *b* comparison. Fitting the full model would thus require a large number of possibly multi-arm studies in order to make **Σ**_(*bk*)_ identifiable [5,13]. The number of parameters in *Σ*_(*bk*)_, can however be reduced if reasonable assumptions can be made about the covariance structure. In particular, most practical applications of NMA methods involve the assumption of a common between-study variance across treatment arms, often referred to as a homogenous variance assumption [18,40,41]. Therefore, to simplify **Σ**_(*bk*)_ we make the additional assumption in this context of a common between-study correlation ($\rho_{\mathrm{bk}}^{\mathrm{mn}}\;=\;\rho^{\mathrm{mn}}$) leading to the following simplified between-study covariance structure for two-arm studies:

(7) $$\begin{array}{rcl} \left(\begin{array}{c} \begin{array}{c} \delta_{i\left(\mathrm{bk}\right)1}\\ \vdots \end{array}\\ \delta_{i\left(\mathrm{bk}\right)M} \end{array}\right) & \sim & \mathrm{Normal}\left(\left(\begin{array}{c} \begin{array}{c} d_{\left(\mathrm{bk}\right)1}=d_{\left(\mathrm{Ak}\right)1}-d_{\left(\mathrm{Ab}\right)1}\\ \vdots \end{array}\\ d_{\left(\mathrm{bk}\right)M}=d_{\left(\mathrm{Ak}\right)M}-d_{\left(\mathrm{Ab}\right)M} \end{array}\right),\Sigma_{\left(M\times M\right)}\right)\\ \Sigma_{\left(M\times M\right)} & = & \left(\begin{array}{ccc} \sigma_{1}^{2} & \cdots & \rho^{1M}\sigma_{1}\sigma_{M}\\ & \ddots & \vdots \\ & & \sigma_{M}^{2} \end{array}\right) \end{array}$$

where, as in the univariate case, *σ*_*m*_ represent the common between-study standard deviation or heterogeneity parameter specific to outcome *m*.

To include multi-arm studies in our model, we extend equations (3) and (4) to the multiple outcome setting. We show in Appendix A, that under evidence consistency and the homogenous between-study covariance structure ($\sigma_{\left(\mathrm{bk}\right)m}^{2}=\sigma_{m}^{2}\;\mathrm{and}\;\rho_{\mathrm{bk}}^{\mathrm{mn}}\;=\;\rho^{\mathrm{mn}}$), equation (3) can be extended to the multiple outcome settings by formulating the distribution of effects in a multi-arm study *i* with *p + 1* arms reporting on *m* = 1, 2, ⋯, *M* outcomes as follows:

(8) $$\begin{array}{l} \left(\begin{array}{c} \left(\begin{array}{c} \delta_{i\left(bk_{1}\right)1}\\ \vdots \\ \delta_{i\left(bk_{1}\right)M} \end{array}\right)\\ \left(\begin{array}{c} \delta_{i\left(bk_{2}\right)1}\\ \vdots \\ \delta_{i\left(bk_{2}\right)M} \end{array}\right)\\ \begin{array}{c} \vdots \\ \left(\begin{array}{c} \delta_{i\left(bk_{p}\right)1}\\ \vdots \\ \delta_{i\left(bk_{p}\right)M} \end{array}\right) \end{array} \end{array}\right)\sim \mathrm{Normal}\left(\left(\begin{array}{c} \left(\begin{array}{c} d_{\left(bk_{1}\right)1}\\ \vdots \\ d_{\left(bk_{1}\right)M} \end{array}\right)\\ \left(\begin{array}{c} d_{\left(bk_{2}\right)1}\\ \vdots \\ d_{\left(bk_{2}\right)M} \end{array}\right)\\ \begin{array}{c} \vdots \\ \left(\begin{array}{c} d_{\left(bk_{p}\right)1}\\ \vdots \\ d_{\left(bk_{p}\right)M} \end{array}\right) \end{array} \end{array}\right),\;\Sigma_{\left(\mathrm{Mp}\times \mathrm{Mp}\right)}\right)\\ \Sigma_{\left(\mathrm{Mp}\times \mathrm{Mp}\right)}=\\ \left(\left(\begin{array}{lll} \left(\begin{array}{ccc} \sigma_{1}^{2} & \cdots & \rho^{1M}\sigma_{1}\sigma_{M}\\ \vdots & \ddots & \vdots \\ \rho^{1M}\sigma_{1}\sigma_{M} & \cdots & \sigma_{M}^{2} \end{array}\right)\frac{1}{2} & \left(\begin{array}{ccc} \sigma_{1}^{2} & \cdots & \rho^{1M}\sigma_{1}\sigma_{M}\\ \vdots & \ddots & \vdots \\ \rho^{1M}\sigma_{1}\sigma_{M} & \cdots & \sigma_{M}^{2} \end{array}\right) & \begin{array}{cc} \cdots & \frac{1}{2}\left(\begin{array}{ccc} \sigma_{1}^{2} & \cdots & \rho^{1M}\sigma_{1}\sigma_{M}\\ \vdots & \ddots & \vdots \\ \rho^{1M}\sigma_{1}\sigma_{M} & \cdots & \sigma_{M}^{2} \end{array}\right) \end{array}\\ & \left(\begin{array}{ccc} \sigma_{1}^{2} & \cdots & \rho^{1M}\sigma_{1}\sigma_{M}\\ \vdots & \ddots & \vdots \\ \rho^{1M}\sigma_{1}\sigma_{M} & \cdots & \sigma_{M}^{2} \end{array}\right) & \vdots \frac{1}{2}\left(\begin{array}{ccc} \sigma_{1}^{2} & \;\cdots & \rho^{1M}\sigma_{1}\sigma_{M}\\ \vdots & \ddots & \vdots \\ \rho^{1M}\sigma_{1}\sigma_{M} & \cdots & \sigma_{M}^{2} \end{array}\right)\\ & & \begin{array}{cc} \ddots & \vdots \\ & \left(\begin{array}{ccc} \sigma_{1}^{2} & \cdots & \rho^{1M}\sigma_{1}\sigma_{M}\\ \vdots & \ddots & \vdots \\ \rho^{1M}\sigma_{1}\sigma_{M} & \cdots & \sigma_{M}^{2} \end{array}\right) \end{array} \end{array}\right)\right) \end{array}$$

where *p* is the number of treatment effect estimates*.* The corresponding marginal and conditional distributions for arm *j*, given the previous 1, ⋯, (*j* − 1) arms are:

(9) $$\begin{array}{ll} \left(\begin{array}{c} \delta_{i\left(bk_{1}\right)1}\\ \vdots \\ \delta_{i\left(bk_{1}\right)M} \end{array}\right)\sim & \mathrm{Normal}\left(\left(\begin{array}{c} d_{\left(bk_{1}\right)1}\\ \vdots \\ d_{\left(bk_{1}\right)M} \end{array}\right),\right)\\ \Sigma_{\left(M\times M\right)}= & \left(\left(\begin{array}{ccc} \sigma_{1}^{2} & \cdots & \rho^{1M}\sigma_{1}\sigma_{M}\\ \vdots & \ddots & \vdots \\ \rho^{M1}\sigma_{1}\sigma_{M} & \cdots & \sigma_{M}^{2} \end{array}\right)\right)\mathrm{for}j=1\\ \left(\begin{array}{c} \delta_{i\left(bk_{j}\right)1}\\ \vdots \\ \delta_{i\left(bk_{j}\right)M} \end{array}\right)| & \left(\begin{array}{c} \left(\begin{array}{c} \delta_{i\left(bk_{1}\right)1}\\ \vdots \\ \delta_{i\left(bk_{1}\right)M} \end{array}\right)\\ \left(\begin{array}{c} \delta_{i\left(bk_{2}\right)1}\\ \vdots \\ \delta_{i\left(bk_{2}\right)M} \end{array}\right)\\ \begin{array}{c} \vdots \\ \left(\begin{array}{c} \delta_{i\left(bk_{j-1}\right)1}\\ \vdots \\ \delta_{i\left(bk_{j-1}\right)M} \end{array}\right) \end{array} \end{array}\right)\sim \mathrm{Normal}\\ & \left(\left(\begin{array}{c} d_{\left(bk_{j}\right)1}+\frac{1}{j}\sum_{t=1}^{j-1}\left(\delta_{i\left(bk_{t}\right)1}-d_{\left(bk_{t}\right)1}\right)\\ \vdots \\ d_{\left(bk_{j}\right)M}+\frac{1}{j}\sum_{t=1}^{j-1}\left(\delta_{i\left(bk_{t}\right)M}-d_{\left(bk_{t}\right)M}\right) \end{array}\right),\Sigma^{'}=\frac{\left(j+1\right)}{2j}\Sigma_{\left(M\times M\right)}\right)\\ & \;\mathrm{for}\;j=2,\;\ldots ,p \end{array}$$

To complete model 2, *μ*_*ibm*_ and *d*_(1*k*)*m*_ are given minimally informative prior distributions:

$$\mu_{\mathrm{ibm}},\;d_{\left(1k\right)m}\;\sim \;\mathrm{Normal}\left(0,\;10^{3}\right)$$

Prior distributions also need to be specified for **Σ**_(*M* × *M*)_ which, in general, is non-trivial because of the positive definite constraint. Initially we specified an Inverse-Wishart distribution [42]:

$$\Sigma_{\left(M\times M\right)}\sim \mathrm{Inverse}-\mathrm{Wishart}\left(K,M\right)$$

where **K** is *M* × *M* scale matrix and *M* is the total number of outcomes. Specifying minimally informative Inverse-Wishart prior distribution is, however, problematic, especially when the amount of data is small relative to the dimensions of **Σ**_(*M* × *M*)_ as is the case for our example data. Therefore, to allow for flexibility in formulating a prior distribution for **Σ**_(*M* × *M*)_, we also followed a strategy outlined by Lu and Ades (2009) [43] and more recently by Wei and Higgins (2013) [39] to express **Σ**_(*M* × *M*)_ in terms of a diagonal matrix of standard deviations V^1/2^ and squared positive semi-definite matrix of correlations **R** based on a separation strategy (Barnard *et al.*[44]):

$$\Sigma_{\left(M\times M\right)}=\;V^{1/2}\;R\;V^{1/2}$$

where the off-diagonal elements of **R** contain correlation terms and diagonal elements equal 1. Lu and Ades [43] and also Wei and Higgins [39] showed that **R** can be written as **R = L**^**T**^**L** using Cholesky decomposition where **L** is an upper triangular matrix. The spherical parameterization technique [39,43] can be used to express **R** in terms of sine and cosine functions of the elements in **L**. Using this later technique, we specified Uniform (0,π) prior distributions for the spherical coordinate *φ*_*mn*_ in our model to ensure that elements of the correlation matrix **R** lie in the interval (−1,1). Finally, the elements of **V**^1/2^ correspond to the between-study standard deviation terms in **Σ**_(*M* × *M*)_ and are given independent Uniform (0,2) prior distributions as in the univariate case (model 1).

### Model 3: Borrowing strength across interventions and outcomes

From Table 1, it can be seen that none of the studies had considered the interventions E + HSI and E + HV for storage of medicines and storage of other household products. Similarly, interventions E + FE + F and FE were not trialled by any of the included studies on possession of a PCC number. To estimate the full set of 24 basic intervention effects relative to usual care from 9 interventions on 3 outcomes, we applied methods originally proposed by DuMouchel and Harris [29] and revisited by DuMouchel and Groer [45] and Jones *et al.*[30]. We assume that the pooled effects of treatment *k* relative to usual care intervention *d*_(*Ak*)*m*_, can be expressed as a sum of treatment-specific effect *α*_*k*_ and outcome-specific effect *γ*_*m*_. This assumption replaces the minimally informative prior distribution N(0, 10^3^) specified for *d*_(*Ak*)*m*_ in model 2 with:

(10) $$\begin{array}{rl} d_{\left(\mathrm{Ak}\right)m} & \sim \;\mathrm{Normal}\left(\alpha_{k}+\gamma_{m},\;\tau^{2}\right)\\ k & =2,\;3,\;\cdots K;\;m=1,2,\;M \end{array}$$

where *K* is the total number of treatments being evaluated across *M* outcomes, and for *k* = 1 (i.e. reference treatment *A*), *d*_(*Ak*)*m*_ equal to zero. Note that on the logarithmic scale, this would imply that the ratio of any intervention effects is constant across outcomes as the *γ*_*m*_ cancel, i.e.

(11) $$d_{\left(\mathrm{bk}\right)m}=\left(d_{\left(\mathrm{Ak}\right)m}-d_{\left(\mathrm{Ab}\right)m}\right)\sim \text{Normal}\left(\alpha_{k}-\alpha_{b},2\tau^{2}\right)$$

Equation (10) thus embodies an assumption of equal or constant relative potency of treatments across outcomes which imply exchangeability of the relative effects between the non-reference/baseline treatments indicated by equation (11). For our example dataset, this implies that missing intervention effects for comparisons with the usual care intervention can be predicted directly from equation (10) as a linear combination of *γ*_*m*_ and *α*_*k*_ assuming that each treatment effect relative to usual care is reported on at least one outcome. The missing intervention effects between non- reference/baseline treatments if required can similarly be predicted directly from the model as linear combinations of the intervention effects relative to usual care.

The parameter *τ* controls the accuracy of the constant relative potency assumption. Values of *τ* close to zero would thus indicate a high degree of confidence (and support from the data) in the parallelism of effect profiles across outcomes and the constant relative potency assumption. Conversely, larger values of *τ* would indicate otherwise.

Multi-arm studies are included in model 3 based on equations (8) and (9) in the same way as in model 2. To complete model 3, the parameters *α*_*k*_, *γ*_*m*_ and *τ* are given minimally informative prior distributions. For the mean effects, this is a normal distribution with zero mean and large variance:

$$\alpha_{k},\;\gamma_{m}\sim \;\mathrm{Normal}\left(0,\;10^{3}\right)$$

We give *τ* a Uniform (0, 2) prior distribution, considered to be minimally informative on the log-odds ratio scale. Sensitivity analyses were conducted to assess the impact of specifying alternative prior distributions for *τ* that are also considered minimally informative [46]:

i. Normal prior distribution centred on 0 with large variance and constrained to be positive, *τ* ~ N(0, 10^2^), *τ* ≥ 0

ii. Gamma prior distribution placed on the precision: *τ*^− 2^ ~ Gamma(0.001, 0.001).

The results of the sensitivity analyses are presented in Additional file 1: Figure S1.

There is a limitation to the number of data (i.e. intervention effects relative to the usual care) on outcomes allowed to be missing for the model hyper-parameters to be identifiable. For *K* interventions and *M* outcomes, there will be (*K* − 1) × *M* equation (10) that are used to estimate a total of (*K* − 1) + *M* hyper-parameters (i.e. (*K* − 1) of *α*_*k*_ and *M* of *γ*_*m*_ hyper-parameters). Therefore no more than ((*K* − 1) × *M*) − ((*K* − 1) + *M*) missing values in total are allowed. For example, for *K* = 3 treatments and *M = 2* outcomes, data has to be available on both outcomes for both treatment comparisons with the baseline when the prior distributions are non-informative. When large number of data on outcomes is missing, placing informative prior distributions on the hyper-parameters can improve convergence.

### Implementation of the models

We fitted a total of four models, models 1 and 3 as described above and two versions of model 2. In model 2a, we specified an inverse-Wishart prior distribution for the between-study covariance matrix **Σ**_(*M* × *M*)_ whilst in model 2b, we specified a prior distribution for **Σ**_(*M* × *M*)_ based on the separation strategy. All four models allowed for multi-arm trials to be included in the analysis. To fit the multivariate NMA models, the quantities $\left({\overset{\hat{\;}}{\theta }}_{\mathrm{ik}1},\;{\overset{\hat{\;}}{\theta }}_{\mathrm{ik}2},\;{\overset{\hat{\;}}{\theta }}_{\mathrm{ik}3}\right)$ and the diagonals of **S**_*ik*_ were estimated using standard 2×2–*table* formulae [2]. Next, we obtained estimates of the within-study correlations from the IPD studies using the following three methods: i) Pearson correlation coefficient between the observed outcome events ii) Bootstrapping as described in Daniel and Hughes (1998), and iii) Generalised Estimating Equations (See details in Additional file 1). All three methods produced identical estimates of the correlations between pairs of outcome specific log-odds of event from each IPD study (Additional file 1: Table S1). Therefore, we formulated informative prior distributions for the correlation terms in **S**_*ik*_ of equation (5) using the estimates of the correlations between the observed outcome events (Pearson correlation) as follows:

$$\begin{array}{l} r_{\mathrm{ik}}^{\mathrm{mn}}\;\sim \;\mathrm{Uniform}\left(a^{\mathrm{mn}},b^{\mathrm{mn}}\right)\;\mathrm{with}\;\\ a^{\mathrm{mn}}=\bar{r^{\mathrm{mn}}}-\left(\frac{\sqrt{12\times \mathrm{var}\left(r^{\mathrm{mn}}\right)}}{2}\;\right)\mathrm{and}\\ b^{\mathrm{mn}}=\bar{r^{\mathrm{mn}}}+\left(\frac{\sqrt{12\times \mathrm{var}\left(r^{\mathrm{mn}}\right)}}{2}\;\right) \end{array}$$

where $r_{\mathrm{ik}}^{\mathrm{mn}}$ is the within-study correlation between the outcomes *m* and *n* effects measured on the log-odds scale in arm *k* of study *i*, and $\bar{r^{\mathrm{mn}}}$ and *var* (*r*^*mn*^)are the mean and variance of the within-study correlation between outcomes *m* and *n* effects measured by IPD respectively.

We also assessed consistency of the evidence within each network using a method based on node splitting [24]. We found no evidence of conflict between the direct and indirect sources (Additional file 1: Table S2) in all three outcome networks.

We fitted all models described above in WinBUGS [47] using Markov Chain Monte Carlo (MCMC) simulations. The univariate models were fitted separately for each outcome using WinBUGS code available from Dias *et al.*[48]. The WinBUGS code for the multivariate models is provided in the appendices 1 and 2 of the Additional file 1. Convergence was assessed by examination of the trace and autocorrelation plots and the Rubin-Gelman statistic after running 400 000 simulations and discarding the first 200 000 samples as ‘burn in samples’.

## Results

### Univariate and multivariate analyses

**Figure 2:**
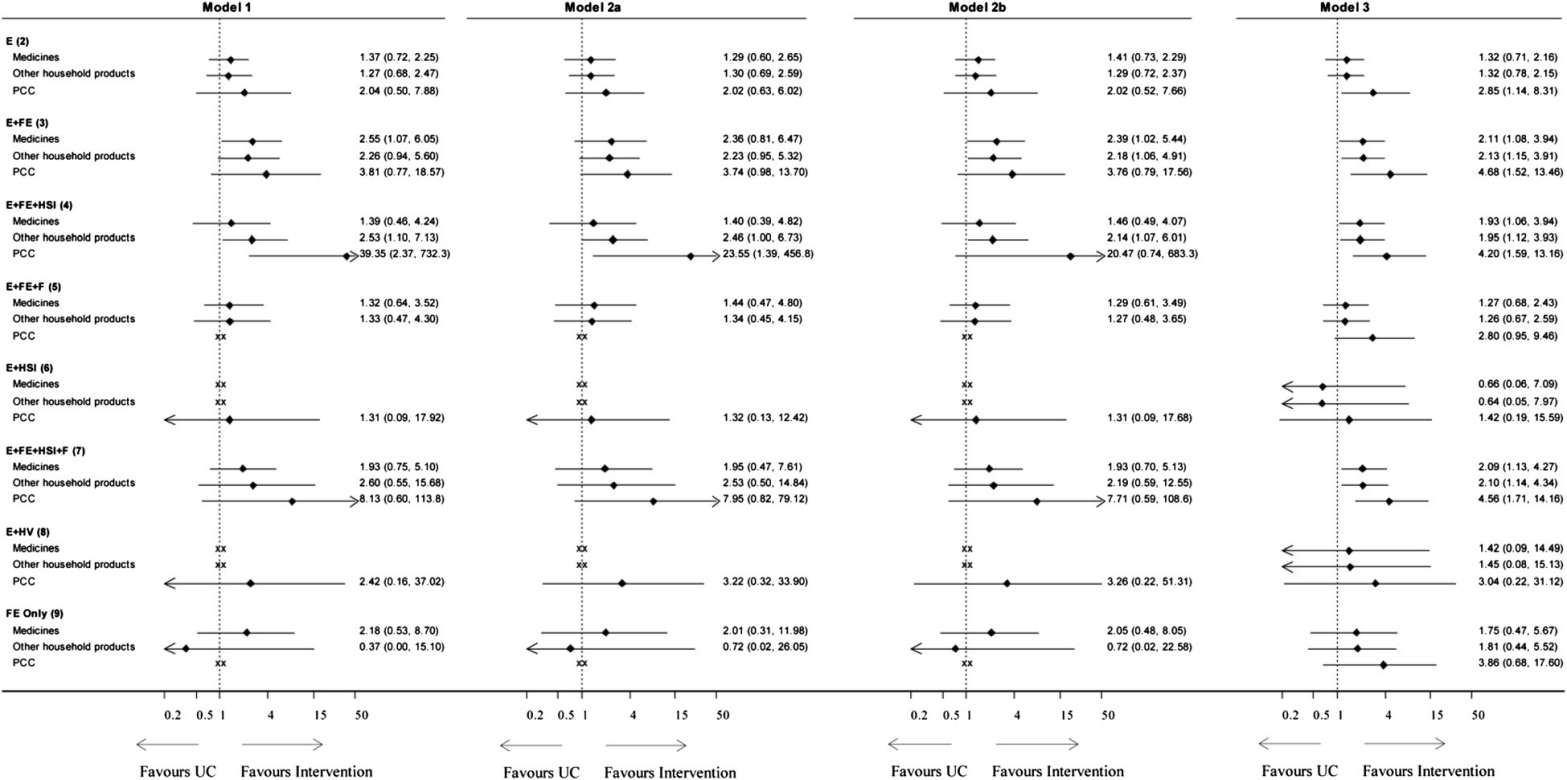
**Summary forest plot of intervention effects relative to usual.** Outcomes are safe storage of medicines, safe storage of other household products and possession of a PCC telephone number. Model 1: Univariate NMA. Model 2a: Multivariate NMA (Wishart prior distribution). Model 2b: Multivariate NMA (separation strategy). Model 3: Multivariate NMA allowing for the relative effects between non-usual care interventions to be exchangeable across outcomes. Effect estimate for which direct study data was not available are indicated by xx on the forest plot. Intervention components: E = Education, FE = low cost/free equipment, HSI = Home safety inspection, HV = Home visit and F= Fitting of equipment.

Parameters of interest were the posterior median estimate (and 95% credible intervals) of the pooled intervention effects relative to the usual care intervention and the posterior median estimate (and 95% credible intervals) of the between-study standard deviation and correlation terms. Summary forest plots displaying effectiveness estimates relative to usual care on the odds ratio (OR) scale are presented in Figure 2. It can be seen that, all 4 models produced broadly similar estimates when the treatment effect is not extreme compared to the other effect estimates for the same outcome. Compared to the univariate analysis, the multivariate models produced noticeably less extreme estimates of intervention effects. This can be seen in the effect of FE + HSI (3) on possession of PCC number being shifted towards the line of no effect from an OR of 39.35 (95% CrI 2.37 to 732.30) in model 1 to 23.55 (95% CrI 1.39, to 456.80) in model 2a, 20.37 (95% CrI 0.72, to 706.00) in model 2b and 4.20 (95% CrI 1.59 to 13.16) in model 3. Similarly, the OR for FE (9) on safe storage of other household products shifted from 0.37 (95% CrI 0.00 to 15.10) in model 1 to 1.81 (95% CrI 0.63, to 5.37) in model 3.

Posterior median and 95% credible intervals of the between-study standard deviations and correlations are presented in Table 2. The posterior medians of the between-study correlations from the multivariate models were small and estimated with considerable uncertainty (i.e. all had large variances). Estimates of the between-study standard deviations were broadly similar for the univariate NMA (model 1) and the multivariate NMA using the separation strategy (model 2b), and relatively high for multivariate NMA using the inverse-Wishart prior distribution (model 2a).

**Table 2:** Posterior median and 95% credible intervals of the between-study standard deviation and correlation parameters

**Parameter**	**Description/prior distribution**	**Model 1: univariate**	**Model 2a: Multivariate using inverse-Wishart prior distribution for Σ**_**(*M*** **×** ***M*)**_	**Model 2b: Multivariate using a separation strategy to specify priors for elements of Σ**_**(*M*** **×** ***M*)**_	**Model 3: Multivariate with extrapolation of effects across outcomes**
*σ*_1_	Between-study standard deviation: safe storage of medicines	0.26 (0.03, 1.02)	0.58 (0.33, 1.18)	0.27 (0.01, 1.08)	0.23 (0.01, 0.80)
*σ*_2_	Between-study standard deviation: safe storage of other household products	0.56 (0.13, 1.27)	0.62 (0.35, 1.15)	0.47 (0.04, 1.18)	0.31 (0.01, 0.81)
*σ*_3_	Between-study standard deviation: PCC	1.16 (0.57, 1.93)	0.94 (0.53, 1.99)	1.18 (0.57, 1.93)	1.08 (0.58, 1.85)
*τ*	Primary analysis: *τ ~*Uniform *(0, 2)*	-	-	-	0.10 (0.01, 0.53)
*τ*	Sensitivity analysis: *τ* ~ Normal (0, 10^2^), *τ* ≥ 0	-	-	-	0.11 (0.00, 0.56)
*τ*	Sensitivity analysis: *τ*^2^ ~ Inverse − Gamma (0.001, 0.001)	-	-	-	0.08 (0.02, 0.36)
*ρ*^12^	Between-study correlation [medicines, other household products]	-	0.03 (−0.73, 0.76)	0.05 (−1.00, 1.00)	0.45 (−0.99, 1.00)
*ρ*^13^	Between-study correlation [medicines, PCC]	-	0.06 (−0.80, 0.81)	0.20 (−1.00, 1.00)	0.50 (−0.98, 1.00)
*ρ*^23^	Between-study correlation [Other household products, PCC]	-	0.08 (−0.81, 0.83)	0.13 (−0.97, 0.98)	0.60 (−0.87, 0.99)

### Borrowing strength across outcomes

It can be seen from Figure 2 that the effect of E + HSI and E + HV relative to usual care intervention on safe storage of medicines and safe storage of other household products, and E + FE + F and FE on possession of a PCC telephone number were only estimated in model 3 as none of the studies had trialled these interventions on the respective outcomes. In this model, estimates of relative effects between non- reference/baseline treatments were assumed to be exchangeable across outcomes, which enabled estimates to be obtained for all outcomes by predicting effects where the interventions have not been considered for the particular outcome of interest. For the intervention/outcome pair where data from trials were available, this extrapolation step had the additional effect of producing more precise estimates of the treatment effect in comparison to the models that do not assume exchangeability effects across outcomes.

The posterior median and 95% credible intervals of intervention effects relative to usual care were unaffected by placing alternative minimally informative prior distributions on *τ* (Additional file 1: Appendix 2). The posterior median and credible intervals for *τ* (Table 2) were similarly not sensitive to the choice of prior distribution placed on *τ* in the primary and sensitivity analyses. The posterior median estimates of *τ* were all close to zero, which suggest that assumptions about the parallelism of effect profiles across outcomes is supported by the data. The estimates of *τ* would thus suggest with 95% probability, that based on the information in our example dataset, the extrapolation model could be accurate to within a factor of about *e*^(2 × 0.10)^ = 1.24 (95% CrI 1.01 to 2.87).

## Discussion

We have presented methods for simultaneous comparison of multiple treatments across multiple outcome measures while preserving the internal randomisation of individual studies. Our method may be viewed as an extension of Ades *et al.’s* (2010) NMA with competing risks paper [13] wherein only the within-study correlation is taken into account. We have extended their method to account for the dependency between outcome effects across studies as well as within-studies.

In this particular application of the multivariate approach, accounting for the correlation between outcomes alone (models 2a and 2b) did not reduce the uncertainty around estimates of intervention effects compared to analysing each outcome separately (model 1). Assuming that intervention effects are exchangeable across outcome did however lead to a modest reduction in uncertainty around effectiveness estimates (model 3).

The between-study correlations were estimated with considerable uncertainty (Table 2) and appear to have little impact on overall effect estimates. This may be because the between-study correlation arises due to, among other things, differences in study-level characteristics that also give rise to between-study heterogeneity in a meta-analysis. Based on a criterion outlined in Spiegelhalter *et al.*[49] the posterior median estimates of the between-study standard deviations, *σ*_1_ and *σ*_2_ on the log odds ratio scale (Table 2) could be interpreted as indicating evidence of low to moderate heterogeneity for storage of medicines and storage of other household products outcomes. Only the estimates for possession of poison control centre number exhibited a considerable degree of heterogeneity. Consequently, the posterior medians of the between-study correlations were small. There was therefore very little gain (in terms of increasing the precision of estimates) from formulating the between-study covariance structure described for the analysis presented here. Accounting for the between-study correlation is likely to be beneficial in situations where the between-study variance (heterogeneity) is large relative to within-study variances.

We opted to incorporate the within-study correlation through the arm-specific effects (log-odds) rather than the study-specific treatment difference (log-odds ratio) as is often done in multivariate meta-analysis [3,4,15,38]. This approach greatly simplifies the likelihood for multi-arm studies because treatment arms can be considered independent as a consequence of randomisation. Hence, there is no requirement to account for the additional correlations between effect estimates which share a common comparator treatment in the model likelihood [50]. The arm-based approach is also likely to be useful when (as is typical with many practical application of multivariate meta-analysis) the within-study correlations are not available [10,12,15,51] and have to be obtained from an external source such as expert opinion [14]. In such situations, formulating questions about correlations between outcome-specific event probabilities (which can be used directly in an arm-based approach) is more likely to be intuitive and easily understood by non-statistician healthcare experts than questions about correlations between intervention effects. It is acknowledged however, that the correlations between the intervention effects if required can easily be obtained from the correlations between the outcomes [14,51].

At the between-study level, we assumed a common correlation structure across treatments in addition to the common variance assumption underlying most practical application of NMA methods. The common correlation assumption implies that if several separate multivariate meta-analyses were conducted with the same outcomes, each with a different set of *k* versus *b* comparison, the assumption is that the between-study correlations would be the same across the different sets of *bk* comparisons. We suggested this structure to simplify the covariance structure and reduce the number of parameters in the model. Appropriateness of such modelling assumptions would need to be considered carefully and assessed when it is feasible to do so.

Initially we specified an inverse-Wishart distribution for the between-study covariance matrix **Σ**_(*M* × *M*)_. However, we believe this prior distribution to be influential due to the small number of studies in our example dataset relative to the number of outcomes. Under these conditions, the inverse-Wishart prior distribution produced upwardly-biased estimates of *σ*_1_ and *σ*_2_ and downward bias in the estimate for *σ*_3_ when compared to the corresponding estimates obtained from the univariate model (Table 2). These findings are consistent with observations in the univariate case where the use of a Gamma prior distribution (which is the univariate analogue of the Inverse-Wishart prior distribution) can lead to an overestimation of the heterogeneity parameter when the true value is close to 0 [46,52]. As an alternative to an inverse-Wishart prior distribution therefore, we followed the spherical decomposition technique suggested by Lu and Ades [43]. This parameterization offered greater flexibility in formulating independent prior distributions for the standard deviation and correlation terms in **Σ**_(*M* × *M*)_.

An obvious limitation to implementation of the multivariate models presented in this paper is the limited availability of data including i) the problem of missing within-study correlations and ii) the requirement for a relatively large number of studies to estimate all model parameters. The problem of missing within-study correlations has traditionally hampered the widespread application of multivariate meta-analysis [7,10,15]. In our example, IPD was available from a proportion of the included studies and we have used correlations estimated from the IPD to formulate informative prior distributions for the within-study model. Alternative approaches to dealing with missing within-study correlations when IPD is not available include: i) using the observed correlation from the summary study-specific effects [12], ii) eliciting information about the correlations from external sources such as clinical experts [14] and iii) specifying ‘vague’ prior distributions for analysis conducted within a Bayesian framework [6].

The second data issue concerns the number of studies needed to estimate the full unstructured between-study covariance matrix presented in equation (6). We anticipate a large number of multi-arm studies reporting across the three outcomes will be needed to identify **Σ**_(*bk*)_ and estimate all model parameters. This can be problematic considering the fact that most applications of network meta-analysis typically include mostly two-arm studies with very small numbers of multi-arm studies. Even with the simplification of the between-study covariance matrix given in equation (5), a relatively large number of studies in comparison to the total number of outcomes being considered may still be needed. We are unable to answer the question of how many studies should be considered large enough for a NMA with multiple outcomes. As a guide, Wei and Higgins [39] recently estimated 15, 27 and 42 studies as a minimum for multivariate pairwise meta-analysis with two, three and four-outcomes respectively. Hence, we believe an even larger number of studies will be required for the NMA with multiple outcomes.

Another limitation of the multivariate models presented here is that they rely on the normal approximation to binomial distribution to incorporate the within-study correlations in the model. The normal approximation frequently fails and may not provide adequate fit to the data in the presence of studies with zero or a small number of events, necessitating use of continuity corrections. We were unable to use the exact binomial distribution as our primary interest was to develop models for summary binary data where outcomes are not mutually exclusive, and where it is not reasonable to assume that within-study correlations are zero so that the likelihood factorises easily as in Arends *et al.*[3]. Further methodological investigations into modelling multivariate summary data that is not normally distributed will therefore be useful. An example is provided in Chu *et al.*[53] where parameterization of the within-study model enabled the special case of diagnostic sensitivity and specificity to be jointly modelled with disease prevalence using a trivariate binomial likelihood. In the interim, an alternative formulation which bypasses the need for approximating normal distributions is to directly model the IPD where this is available. This will require extending Saramago *et al.’s*[54] NMA model with aggregate and individual participant level data from single outcome to multiple outcome settings.

We assessed the consistency of each outcome network separately using the method of node splitting [24]. We found no evidence of conflict between the direct and indirect sources on pairwise contrasts that have both sources of evidence in model 1. We did not assess the consistency of the multivariate estimates partly because we are unaware of current methods for carrying out this type of assessment. We are investigating extensions of the node-split method to multiple outcome networks and investigate the effect of jointly synthesising evidence across multiple endpoints on evidence consistency in a simulation study.

Our initial motivation for a multiple outcome NMA was to estimate intervention effects for all the outcomes, including effects of interventions on outcomes not considered by any of the studies included in the analysis. This requires the correlation structure between effects on multiple outcomes to be appropriately modelled and also ensuring the mechanism of “borrow strength” across outcomes through the assumption of exchangeability of the random effect across outcomes. This implies a priori assumption that outcomes are related but different and that there is no way of knowing the order of magnitude of effects on outcomes. If this assumption does not hold, it may potentially lead to worse or more biased effectiveness estimates. In our example, the outcomes are similar and measured on the same scale. It would be clearly inappropriate to assume that intervention effects are exchangeable across outcomes that are different in some important respects such as being measured on different scales (e.g. where one outcome reports a weighted mean difference and another outcome reports a log-odds ratio) as such estimates will differ in terms of the precision with which they are estimated.

## Conclusion

Our aim in this paper was to present methods for simultaneous comparison of multiple treatments across multiple outcome measures while preserving the internal randomisation of individual studies. Application of the method to the poison prevention data yielded similar point estimates of treatment effect to those obtained from a univariate NMA but the uncertainty around the multivariate estimates increased or decreased depending on the prior distribution specified for the between-study covariance structure. The proposed method followed the usual hierarchical approach to multivariate meta-analysis where correlations between outcomes are modelled at the within-study and or between-study levels.

## Between-study covariance for multi-arm studies reporting multiple outcomes

For a multi-arm study *i* with *K* treatments labelled *A, B, C, …, K* reporting a total of *M* outcomes labelled *1, 2, …, M*. A random effects between-study model can be represented as:

(A1) $$\left(\begin{array}{c} \begin{array}{c} \left(\begin{array}{c} \delta_{i\left(\mathrm{AB}\right)1}\\ \vdots \\ \delta_{i\left(\mathrm{AB}\right)M} \end{array}\right)\\ \left(\begin{array}{c} \delta_{i\left(\mathrm{AC}\right)1}\\ \vdots \\ \delta_{i\left(\mathrm{AC}\right)M} \end{array}\right) \end{array}\\ \begin{array}{c} \vdots \\ \left(\begin{array}{c} \delta_{i\left(\mathrm{AK}\right)1}\\ \vdots \\ \delta_{i\left(\mathrm{AK}\right)M} \end{array}\right) \end{array} \end{array}\right)\sim \text{Normal}\left(\left(\begin{array}{c} \begin{array}{c} \left(\begin{array}{c} d_{i\left(\mathrm{AB}\right)1}\\ \vdots \\ d_{i\left(\mathrm{AB}\right)M} \end{array}\right)\\ \left(\begin{array}{c} d_{i\left(\mathrm{AC}\right)1}\\ \vdots \\ d_{i\left(\mathrm{AC}\right)M} \end{array}\right) \end{array}\\ \begin{array}{c} \vdots \\ \left(\begin{array}{c} d_{i\left(\mathrm{AK}\right)1}\\ \vdots \\ d_{i\left(\mathrm{AK}\right)M} \end{array}\right) \end{array} \end{array}\right),\Sigma_{\mathrm{FULL}}\right)$$

$$\begin{array}{cccc} \Sigma_{\mathrm{FULL}}=\left(\begin{array}{ccc} \sigma_{\left(\mathrm{AB}\right)1}^{2} & \cdots & \rho_{\left(\mathrm{AB},\mathrm{AB}\right)}^{1M}\sigma_{\left(\mathrm{AB}\right)1}\sigma_{\left(\mathrm{AB}\right)M}\\ \vdots & \ddots & \vdots \\ \rho_{\left(\mathrm{AB},\mathrm{AB}\right)}^{1M}\sigma_{\left(\mathrm{AB}\right)1}\sigma_{\left(\mathrm{AB}\right)M} & \cdots & \sigma_{\left(\mathrm{AB}\right)M}^{2} \end{array}\right) & \left(\begin{array}{ccc} \rho_{\left(\mathrm{AB},\mathrm{AC}\right)}^{11}\sigma_{\left(\mathrm{AB}\right)1}\sigma_{\left(\mathrm{AC}\right)1} & \cdots & \rho_{\left(\mathrm{AB},\mathrm{AC}\right)}^{1M}\sigma_{\left(\mathrm{AB}\right)1}\sigma_{\left(\mathrm{AC}\right)M}\\ \vdots & \ddots & \vdots \\ \rho_{\left(\mathrm{AB},\mathrm{AC}\right)}^{1M}\sigma_{\left(\mathrm{AB}\right)1}\sigma_{\left(\mathrm{AC}\right)M} & \cdots & \rho_{\left(\mathrm{AB},\mathrm{AC}\right)}^{\mathrm{MM}}\sigma_{\left(\mathrm{AB}\right)M}\sigma_{\left(\mathrm{AC}\right)M} \end{array}\right) & \cdots & \left(\begin{array}{ccc} \rho_{\left(\mathrm{AB},\mathrm{AK}\right)}^{11}\sigma_{\left(\mathrm{AB}\right)1}\sigma_{\left(\mathrm{AK}\right)1} & \cdots & \rho_{\left(\mathrm{AB},\mathrm{AK}\right)}^{1M}\sigma_{\left(\mathrm{AB}\right)1}\sigma_{\left(\mathrm{AK}\right)M}\\ \vdots & \ddots & \vdots \\ \rho_{\left(\mathrm{AB},\mathrm{AK}\right)}^{1M}\sigma_{\left(\mathrm{AB}\right)1}\sigma_{\left(\mathrm{AK},\mathrm{AK}\right)M} & \cdots & \rho_{\left(\mathrm{AB},\mathrm{AK}\right)}^{\mathrm{MM}}\sigma_{\left(\mathrm{AB}\right)M}\sigma_{\left(\mathrm{AK}\right)M} \end{array}\right)\\ & \left(\begin{array}{ccc} \sigma_{\left(\mathrm{AC}\right)1}^{2} & \cdots & \rho_{\left(\mathrm{AC},\mathrm{AC}\right)}^{1M}\sigma_{\left(\mathrm{AC}\right)1}\sigma_{\left(\mathrm{AC}\right)M}\\ \vdots & \ddots & \vdots \\ \rho_{\left(\mathrm{AC},\mathrm{AC}\right)}^{1M}\sigma_{\left(\mathrm{AC}\right)1}\sigma_{\left(\mathrm{AC}\right)M} & \cdots & \sigma_{\left(\mathrm{AC}\right)M}^{2} \end{array}\right) & \vdots & \left(\begin{array}{ccc} \rho_{\left(\mathrm{AC},\mathrm{AK}\right)}^{11}\sigma_{\left(\mathrm{AC}\right)1}\sigma_{\left(\mathrm{AK}\right)1} & \;\cdots & \rho_{\left(\mathrm{AC},\mathrm{AK}\right)}^{1M}\sigma_{\left(\mathrm{AC}\right)1}\sigma_{\left(\mathrm{AK}\right)M}\\ \vdots & \;\ddots & \vdots \\ \rho_{\left(\mathrm{AC},\mathrm{AK}\right)}^{1M}\sigma_{\left(\mathrm{AC}\right)1}\sigma_{\left(\mathrm{AC}\right)M} & \;\cdots & \rho_{\left(\mathrm{AC},\mathrm{AK}\right)}^{\mathrm{MM}}\sigma_{\left(\mathrm{AC}\right)M}\sigma_{\left(\mathrm{AK}\right)M} \end{array}\right)\\ & & \ddots & \vdots \\ & & & \left(\begin{array}{ccc} \sigma_{\left(\mathrm{AK}\right)1}^{2} & \cdots & \rho_{\left(\mathrm{AK},\mathrm{AK}\right)}^{1M}\sigma_{\left(\mathrm{AK}\right)1}\sigma_{\left(\mathrm{AK}\right)M}\\ \vdots & \ddots & \vdots \\ \rho_{\left(\mathrm{AK},\mathrm{AK}\right)}^{1M}\sigma_{\left(\mathrm{AK}\right)1}\sigma_{\left(\mathrm{AK}\right)M} & \cdots & \sigma_{\left(\mathrm{AK}\right)M}^{2} \end{array}\right) \end{array}$$

Where *δ*_*i*(*Ak*)*m*_ and *d*_(*Ak*)*m*_ are study-specific and mean effect of treatment *k* relative *A* (reference treatment) on outcome *m* in study *i* respectively and **Σ**_**FULL**_ is the full (K-1) × (K-1) blocks of *M* × *M* within-treatment between-outcome covariance matrix. The parameters in **Σ**_**FULL**_ have the following interpretation:($\sigma_{\left(\mathrm{Ak}\right)m}^{2}$) indicate the variance of the effect of treatment *k* (*k* = *B*, *C*, ⋯, *K*) relative to *A* on outcome *m* across studies.

$\rho_{\left(\mathrm{Ak},\mathrm{Ak}\right)}^{\mathrm{mn}}$indicate the correlation between *δ*_*i*(*Ak*)*m*_ and *δ*_*i*(*Ak*)*n*_ (i.e. the correlation between the effect of treatment *k* relative to *A* on outcome *m* and the effect of treatment *k* relative to *A* on outcome *n* (*m* ≠ *n*)) specific to the *Ak* comparison.

$\rho_{\left(\mathrm{Ah},\mathrm{Ak}\right)}^{\mathrm{mm}}$indicate the correlation between *δ*_*i*(*Ah*)*m*_ and *δ*_*i*(*Ak*)*m*_ (i.e. the correlation between the effect of treatment *h* relative to *A* on outcome *m* and the effect of treatment *k* relative to *A* (*h* ≠ *k*) on outcome *m* because they share a common comparator *A*).The diagonal block matrices in **Σ**_**FULL**_ thus carry terms for the between-study variance ($\sigma_{\left(\mathrm{Ak}\right)m}^{2}$) while the off-diagonal blocks carry terms for the between-study covariance. We make two assumptions to simplify and reduce the number of parameters in **Σ**_**FULL**_. First, we assume homogenous variances for intervention effects within outcomes [20]. This implies $\sigma_{\left(\mathrm{Ak}\right)m}^{2}=\sigma_{m}^{2}$ and $\rho_{\left(\mathrm{Ah},\mathrm{Ak}\right)}^{\mathrm{mm}}=\;\frac{1}{2}$ as in the single outcome network meta-analysis case [20,34]. Second, we make the assumption of homogenous between-study correlations for the intervention effects from different outcomes. Under this assumption we can express $\rho_{\left(\mathrm{Ah},\mathrm{Ah}\right)}^{\mathrm{mn}}$ and $\rho_{\left(\mathrm{Ak},\mathrm{Ak}\right)}^{\mathrm{mn}}$ in terms of a common correlation parameter *ρ*^*mn*^ by noting that for any 3-treatment **(***A, h, k***)** configuration, the covariance between outcome *m* and *n* effects across studies can be expressed as a covariance between two sums under evidence consistency:

(A2) $$\begin{array}{lll} \left[\delta_{i\left(\mathrm{hk}\right)m},\delta_{i\left(\mathrm{hk}\right)n}\right] & = & \mathrm{COV}\left[\left(\delta_{i\left(\mathrm{Ak}\right)m}-\delta_{i\left(\mathrm{Ah}\right)m}\right),\left(\delta_{i\left(\mathrm{Ak}\right)n}-\delta_{i\left(\mathrm{Ah}\right)n}\right)\right]\\ & = & \mathrm{COV}\left[\delta_{i\left(\mathrm{Ak}\right)m},\delta_{i\left(\mathrm{Ak}\right)n}\right]-\mathrm{COV}\left[\delta_{i\left(\mathrm{Ak}\right)m},\delta_{i\left(\mathrm{Ah}\right)n}\right]\\ & & -\mathrm{COV}\left[\delta_{i\left(\mathrm{Ah}\right)m},\delta_{i\left(\mathrm{Ak}\right)n}\right]+\mathrm{COV}\left[\delta_{i\left(\mathrm{Ah}\right)m},\delta_{i\left(\mathrm{Ah}\right)n}\right]\\ & = & \left(\rho_{\left(\mathrm{Ak},\mathrm{Ak}\right)}^{\mathrm{mn}}+\rho_{\left(\mathrm{Ah},\mathrm{Ah}\right)}^{\mathrm{mn}}-2\rho_{\left(\mathrm{Ak},\mathrm{Ah}\right)}^{\mathrm{mn}}\right)\sigma_{m}\sigma_{n} \end{array}$$

The homogenous between-study correlation assumption implies $\rho_{\left(\mathrm{Ah},\mathrm{Ah}\right)}^{\mathrm{mn}}=\;\rho_{\left(\mathrm{Ak},\mathrm{Ak}\right)}^{\mathrm{mn}}=\rho^{\mathrm{mn}}$ and $\rho_{\left(\mathrm{Ak},\mathrm{Ah}\right)}^{\mathrm{mn}}=\;\frac{1}{2}\rho^{\mathrm{mn}}$ for the inequality $-1\leq \left(\rho_{\left(\mathrm{Ak},\mathrm{Ak}\right)}^{\mathrm{mn}}+\rho_{\left(\mathrm{Ah},\mathrm{Ah}\right)}^{\mathrm{mn}}-2\rho_{\left(\mathrm{Ak},\mathrm{Ah}\right)}^{\mathrm{mn}}\right)\;\leq 1$ to hold. Substituting these expressions into equation (A1), we see that the between-study correlation terms equal *ρ*^*mn*^ in the diagonal block of matrices and $\frac{1}{2}\rho^{\mathrm{mn}}$ in the off-diagonal block of matrices of in **Σ**_**FULL**_ leading to the following simplificationfollowing simplification of the between-study covariance matrix:

(A3) $$\begin{array}{l} \left(\begin{array}{c} \begin{array}{c} \left(\begin{array}{c} \delta_{i\left(\mathrm{AB}\right)1}\\ \vdots \\ \delta_{i\left(\mathrm{AB}\right)M} \end{array}\right)\\ \left(\begin{array}{c} \delta_{i\left(\mathrm{AC}\right)1}\\ \vdots \\ \delta_{i\left(\mathrm{AC}\right)M} \end{array}\right) \end{array}\\ \begin{array}{c} \vdots \\ \left(\begin{array}{c} \delta_{i\left(\mathrm{AK}\right)1}\\ \vdots \\ \delta_{i\left(\mathrm{AK}\right)M} \end{array}\right) \end{array} \end{array}\right)\sim \mathrm{Normal}\left(\begin{array}{c} \begin{array}{c} \left(\begin{array}{c} d_{\left(\mathrm{AB}\right)1}\\ \vdots \\ d_{\left(\mathrm{AB}\right)M} \end{array}\right)\\ \left(\begin{array}{c} d_{\left(\mathrm{AC}\right)1}\\ \vdots \\ d_{\left(\mathrm{AC}\right)M} \end{array}\right) \end{array}\\ \begin{array}{c} \vdots \\ \left(\begin{array}{c} d_{\left(\mathrm{AK}\right)1}\\ \vdots \\ d_{\left(\mathrm{AK}\right)M} \end{array}\right) \end{array} \end{array},\Sigma_{\left(\mathrm{Mp}\times \mathrm{Mp}\right)}\right)\\ \begin{array}{cc} \mathrm{with} & \Sigma_{\left(\mathrm{Mp}\times \mathrm{Mp}\right)}=\left(\begin{array}{lllll} \left(\begin{array}{ccc} \sigma_{1}^{2} & \cdots & \rho^{1M}\sigma_{1}\sigma_{M}\\ \vdots & \ddots & \vdots \\ \rho^{1M}\sigma_{1}\sigma_{M} & \cdots & \sigma_{M}^{2} \end{array}\right)\frac{1}{2} & \left(\begin{array}{ccc} \sigma_{1}^{2} & \cdots & \rho^{1M}\sigma_{1}\sigma_{M}\\ \vdots & \ddots & \vdots \\ \rho^{1M}\sigma_{1}\sigma_{M} & \cdots & \sigma_{M}^{2} \end{array}\right) & \cdots & \frac{1}{2} & \left(\begin{array}{ccc} \sigma_{1}^{2} & \cdots & \rho^{1M}\sigma_{1}\sigma_{M}\\ \vdots & \ddots & \vdots \\ \rho^{1M}\sigma_{1}\sigma_{M} & \cdots & \sigma_{M}^{2} \end{array}\right)\\ & \left(\begin{array}{ccc} \sigma_{1}^{2} & \cdots & \rho^{1M}\sigma_{1}\sigma_{M}\\ \vdots & \ddots & \vdots \\ \rho^{1M}\sigma_{1}\sigma_{M} & \cdots & \sigma_{M}^{2} \end{array}\right)\vdots & & \frac{1}{2} & \left(\begin{array}{ccc} \sigma_{1}^{2} & \cdots & \rho^{1M}\sigma_{1}\sigma_{M}\\ \vdots & \ddots & \vdots \\ \rho^{1M}\sigma_{1}\sigma_{M} & \cdots & \sigma_{M}^{2} \end{array}\right)\\ & & & & \left(\begin{array}{ccc} \sigma_{1}^{2} & \cdots & \rho^{1M}\sigma_{1}\sigma_{M}\\ \vdots & \ddots & \vdots \\ \rho^{1M}\sigma_{1}\sigma_{M} & \cdots & \sigma_{M}^{2} \end{array}\right) \end{array}\right) \end{array} \end{array}$$

Finally by relabeling the reference treatment *A* as *b*, (*δ*_*i*(*AB*)1_, ⋯, *δ*_*i*(*AK*)*m*_) as $\left(\delta_{i\left(bk_{1}\right)1},\;\cdots ,\delta_{i\left(bk_{j}\right)M}\;\right)$ and (*d*_(*AB*)1_, ⋯, *d*_(*AK*)*M*_) as $\left(d_{\left(bk_{1}\right)M},\;\cdots ,d_{\left(bk_{j}\right)M}\;\right)$, equation (A3) can be rewritten as equation (A4).

(A4) $$\left(\begin{array}{c} \left(\begin{array}{c} \delta_{i\left(bk_{1}\right)1}\\ \vdots \\ \delta_{i\left(bk_{1}\right)M} \end{array}\right)\\ \left(\begin{array}{c} \delta_{i\left(bk_{2}\right)1}\\ \vdots \\ \delta_{i\left(bk_{2}\right)M} \end{array}\right)\\ \begin{array}{c} \vdots \\ \left(\begin{array}{c} \delta_{i\left(bk_{p}\right)1}\\ \vdots \\ \delta_{i\left(bk_{p}\right)M} \end{array}\right) \end{array} \end{array}\right)\sim \mathrm{Normal}\left(\left(\begin{array}{c} \left(\begin{array}{c} d_{\left(bk_{1}\right)1}\\ \vdots \\ d_{\left(bk_{1}\right)M} \end{array}\right)\\ \left(\begin{array}{c} d_{\left(bk_{2}\right)1}\\ \vdots \\ d_{\left(bk_{2}\right)M} \end{array}\right)\\ \begin{array}{c} \vdots \\ \left(\begin{array}{c} d_{\left(bk_{p}\right)1}\\ \vdots \\ d_{\left(bk_{p}\right)M} \end{array}\right) \end{array} \end{array}\right),\;\Sigma_{\left(\mathrm{Mp}\times \mathrm{Mp}\right)}\right)$$

## Abbreviations

CrI: Credible interval; IPD: Individual patient data; MCMC: Markov Chain Monte Carlo; NMA: Network meta-analysis; PCC: Poison centre control number.

## Competing interests

The authors declare that they have no competing interests.

## Author’s contribution

FAA drafted the initial manuscript, conducted the analysis and coordinated contributions from all authors in drafting the final manuscript. NJC designed the study and revised the manuscript to improve general readability. SB contributed to developing the statistical analysis and interpretation of the results. SJH contributed to preparing the data for analysis, drafting the network diagrams and revision of the initial and final drafts. DK provided the data for analysis, defined the outcomes and interventions and revision of the final drafts.DRJ participated in designing the study and revision of the final manuscript. AJS conceived the original idea and participated developing the statistical model. All authors read and approved the final manuscript.

## Pre-publication history

The pre-publication history for this paper can be accessed here:

http://www.biomedcentral.com/1471-2288/14/92/prepub

## Supplementary Material

Additional file 1 (WinBUGS Code for model 2): Network meta-analysis of multiple outcome measures accounting for borrowing of information across outcomes. Click here for file
