# Supplementary material for: Network meta-analysis of multiple outcome measures accounting for borrowing of information across outcomes
Source: BMC Med Res Methodol. 2014 Jul 21;14:92. doi: 10.1186/1471-2288-14-92 (PMC4142066; doi:10.1186/1471-2288-14-92)
Supplement: Additional file 1 — (WinBUGS Code for model 2): Network meta-analysis of multiple outcome measures accounting for borrowing of information across outcomes. [file 1471-2288-14-92-S1.docx]

**Supplementary material (WinBUGS Code for model 2): Network meta-analysis across multiple outcome measures accounting for borrowing of information across outcomes.**

Authors: Felix Achana^1^, Nicola Cooper^1^, Sylwia Bujkiewicz^1^, Stephanie Hubbard^1^, Denise Kendrick^2^, David Jones^1^, Alex Sutton^1^

Table S1: Within-study correlations between pairs of log-odds ratios estimated from IPD studies

| Study | Pearson correlation coefficient^†^ | | |  | GEE Model^‡^ | | |  | Bootstrap^+^ | | |
| --- | --- | --- | --- | --- | --- | --- | --- | --- | --- | --- | --- |
|  | $r^{12}$ | $r^{13}$ | $r^{23}$ |  | $r^{12}$ | $r^{13}$ | $r^{23}$ |  | $r^{12}$ | $r^{13}$ | $r^{23}$ |
| Gielen 2007* |  |  |  |  |  |  |  |  |  |  |  |
| Nansel 2002 | 0.212 | -0.115 | 0.072 |  | 0.212 | -0.115 | 0.072 |  | 0.204 | -0.000 | 0.104 |
| Nansel 2008 | 0.235 | 0.013 | -0.016 |  | 0.235 | 0.013 | -0.016 |  | 0.234 | 0.010 | -0.013 |
| Clamp 1998 | 0.114 |  |  |  | 0.114 |  |  |  | 0.120 |  |  |
| Babul 2007** |  |  |  |  |  |  |  |  |  |  |  |
| Hendrickson 2002 |  |  |  |  |  |  | 0.458 |  |  |  |  |
| Kendrick 1999** |  |  |  |  |  |  |  |  |  |  |  |
| Watson 2005 | 0.235 |  |  |  | 0.235 |  |  |  | 0.197 |  |  |
| Posner 2004 | 0.324 | -0.055 | 0.096 |  | 0.324 | -0.055 | 0.096 |  | 0.396 | -0.055 | 0.089 |
| Sznajder 2003 | -0.014 |  |  |  | -0.014 |  |  |  | -0.0004 |  |  |
| **Mean (SE)** | **0.184 (0.118)** | **-0.052 (0.064)** | **0.051 (0.059)** |  | **0.184 (0.118)** | **-0.052 (0.064)** | **0.153 (0.209)** |  | **0.192 (0.131)** | **-0.015 (0.035)** | **0.06 (0.064)** |
| †Pearson correlation = correlation between observed outcome events obtained using **pwcorr** command in Stata.  ‡GEE Model with unstructured correlation structure fitted in Stata using **xtgee** command (code given below).  +Bootstrap code given below.  *Gielen 2007, different sets of patients reported safe storage of medicines and safe storage of other household products, hence unable to estimate correlation from this IPD.  **Babul (2007) reported only safe storage of medicines, Kendrick (1999) reported only safe storage of other household products  Outcome1 = Safe storage of medicines  Outcome2 = Safe storage of other household products  Outcome3 = Possession of a poison centre telephone number | | | | | | | | | | | |

**Stata code to estimate correlations between log-odds ratios**

** **Stata code**

// Pearson correlation

pwcorr y1 y2

// reshape from wide to long and fit GEE

qui reshape long y, i(id) j(outcome)

// create indicator variables for each outcome

tab outcome, gen(s)

// Interaction terms for the treatment effect if required

forvalues i=1/2 {

gen x1_`i' = s`i'*t

}

// xtset data and fit GEE with independent correlations

xtset id outcome

// log-odds model include outcome indicator as covariate

xtgee y s1 s2, nocons i(id) link(logit) family(binom)

estat vce, cor

// log-odds ratio model include outcome indicator and interaction terms as covariate

xtgee y s1 s2 x1_1 x1_2, nocons i(id) link(logit) family(binom) corr(uns) robust

estat vce, cor

**Data**

**id=subject id, t=treatment group indicator, y1,y2= outcome 1 and 2

| id[] | t | y1 | y2 |
| --- | --- | --- | --- |
| 1 | 1 | 1 | 1 |
| 2 | 1 | 1 | 1 |
| 3 | 1 | 1 | 0 |
| 4 | 1 | 1 | 1 |
| 5 | 1 | 1 | 1 |
| . | . | . | . |
| . | . | . | . |
| END |  |  |  |

**R code to simulate correlated binary data**

# Parameters of joint distribution

size <- 1

prob<-c(0.5,0.5)

rho<- 0.4

# A function to simulate n correlated pairs

require(bindata)

rmvBinomial <- function(n, size, prob, rho) {

y <- replicate(n, {

colSums(rmvbin(size, prob, bincorr=(1-rho)*diag(2)+rho))

})

t(y)

}

# Create 100 sample size

n <- 100

set.seed(12345)

y <- rmvBinomial(n=n, size=size, prob=prob, rho=rho)

**# Bootstrap to estimate correlation between pair of log-odds**

Nb<-10000

lodds1<-lodds2<-array(0,dim=Nb)

p1<-p2<-c(rep(0,Nb))

s<-seq(1:n)

Nb<-10000

lodds1<-lodds2<-array(0,dim=Nb)

p1<-p2<-c(rep(0,Nb))

for (i in 1:Nb){

sam<-sample(s, replace=T)

new_out1<-y[sam,1]

new_out2<-y[sam,2]

p1[i]<-mean(new_out1)

p2[i]<-mean(new_out2)

lodds1[i]<-log(p1[i]/(1-p1[i]))

lodds2[i]<-log(p2[i]/(1-p2[i]))

}

#pairwise correlations

cor(lodds1,lodds2) # bootstrap estimate

cor(y[,1],y[,2], method=”pearson”) #Pearson correlation estimate

Table S2: Posterior means and standard deviations (sd) of the log-odds ratios using the full network, direct and indirect evidence on each pairwise comparison

| Pair-wise contrast | Combined evidence from NMA model | Direct evidence | Indirect evidence | Inconsistency estimate^†^ | p-value* |
| --- | --- | --- | --- | --- | --- |
| **Safe storage of medicines** |  |  |  |  |  |
| Usual care (1) vs. Education (2) | 0.40 (0.30) | 0.50 ( 0.34) | 0.79 (0.86) | 0.89 ( 0.97) | 0.306 |
| Usual care (1) vs. Education + Free/low cost Equipment (3) | 0.74 (0.61) | 0.48 (1.23) | 0.38 (0.36) | -0.31 (1.46) | 0.820 |
| Usual care (1) vs. Education + Equipment + Fitting (5) | 0.35 (0.49) | 0.14 (0.56) | 1.55 (1.15) | -1.41 (1.27) | 0.232 |
| Education (2) vs. Education + Free/low cost Equipment (3) | 0.34 (0.58) | 0.41 (0.79) | 0.12 (1.20) | 0.29 (1.44) | 0.778 |
| Education (2) vs. Education + Equipment + Fitting (5) | -0.05 (0.54) | 1.24 (1.09) | -0.32 (0.68) | 1.56 (1.27) | 0.189 |
| **Safe storage of other household products** |  |  |  |  |  |
| Usual care (1) vs. Education (2) | 0.24 (0.32) | 0.36 (0.38) | -0.24 (0.73) | 0.61 (0.83) | 0.408 |
| Usual care (1) vs. Education + Free/low cost Equipment (3) | 0.82 (0.43) | 0.68 (0.54) | 1.29 (0.91) | -0.61 (1.05) | 0.499 |
| Usual care (1) vs. Education + Equipment + Fitting (5) | 0.30 (0.54) | 0.17 (0.76) | 0.60 (1.01) | -0.43 (1.27) | 0.698 |
| Education (2) vs. Education + Equipment (3) | 0.58 (0.48) | 0.96 (0.85) | 0.34 (0.67) | 0.61 (1.07) | 0.508 |
| Education (2) vs. Education + Equipment + Fitting (5) | 0.07 (0.56) | 0.31 (0.93) | -0.12 (0.83) | 0.43 (1.25) | 0.690 |
| Education + Equipment (3) vs. Education + Equipment + Home safety inspection (4) |  |  |  |  |  |
| **Possession of a PCC number** |  |  |  |  |  |
| Usual care (1) vs. Education (2) | 0.71 (0.68) | 0.70 (0.81) | 0.72 (1.72) | -0.02 (1.89) | 0.989 |
| Usual care (1) vs. Education + Equipment (3) | 1.33 (0.78) | 1.34 (0.97) | 0.69 (0.81) | 0.04 (1.90) | 0.986 |
| Education (2) vs. Education + Equipment (3) | 0.63 (0.879) | 0.63 (1.43) | 0.63 (1.26) | 0.00 (1.91) | 0.992 |
| †inconsistency estimate = direct estimate – indirect estimate of the treatment effect (log-OR)  *p-value = $2\times\left( probability of direct estimate > indirect estimate \right)$ which gives the 2-sided probabilities that the direct and indirect evidence are different | | | | | |

Favours UC Favours Intervention

Favours UC Favours Intervention

Favours UC Favours Intervention

**Figure S1:** Results of sensitivity analysis to different specifications of prior distributions for tau($\tau$) in Model 3. Intervention components: E = Education, FE=Free equipment, HSI = Home safety inspection, HV = Home visit and F= Fitting of equipment. IG = Inverse-Gamma distribution, N=Normal distribution and U = Uniform distribution

Odds ratio and 95% credible intervals in brackets (log-scale)

**Appendix 1: WinBUGS code for model 2 code**

Model { #model 2b

# i = data point (one for each arm of each study),

# arm = study arm

# s = study

# m = outcome

**#Likelihood for arm level data**

**#=======================**

for(i in 1:N1){

tmp1[i] <- studyid[i] # study id not used in the model

y[i,1:3] ~ dmnorm(mean.y[study[i],arm[i],1:3],omega[i,,]) # multivariate likelihood

omega[i,1:3,1:3] <- inverse(cov.mat[i,,]) # within-study precision matrix

#define elements of within-study covariance matrix

cov.mat[i,1,1] <- pow(se[i,1],2)

cov.mat[i,2,2] <- pow(se[i,2],2)

cov.mat[i,3,3] <- pow(se[i,3],2)

cov.mat[i,1,2] <- se[i,1]*se[i,2]*cor[i,1]

cov.mat[i,1,3] <- se[i,1]*se[i,3]*cor[i,2]

cov.mat[i,2,3] <- se[i,2]*se[i,3]*cor[i,3]

cov.mat[i,2,1] <- cov.mat[i,1,2]

cov.mat[i,3,1] <- cov.mat[i,1,3]

cov.mat[i,3,2] <- cov.mat[i,2,3]

for(m in 1:no){

se[i,m] ~ dnorm(0, prec.se[m])I(0,) # input missing standard errors

unif.a[i,m] <- mn.rhoW[m] - (sqrt(12)*se.rhoW[m]/2) # parameter a of uniform distribution

unif.b[i,m] <- mn.rhoW[m] +(sqrt(12)*se.rhoW[m]/2) # parameter b of uniform distribution

cor[i,m] ~ dunif(unif.a[i,m], unif.b[i,m]) # within-study correlation model

}

}

for(j in 1:ns){

for(k in 1:NA[j]) **{**

for(m in 1:no){

mean.y[j,k,m] <- mu[j,m] + delta[j,k,m] # define study-specific treatment effects

}

}

**}**

**#Random effects between-study model**

**#=================================**

for(j in 1:ns) {

for(m in 1:no) {

delta[j,1,m] <-0 # delta in control arm to zero for all outcomes

w[j,1,m] <-0 # multi-arm adjustment in control group set to zero

}

for(k in 2:NA[j]){

delta[j,k,1:no] ~ dmnorm(md[j,k,1:no],precBK[j,k,1:no,1:no]) #random effects model

for(m in 1:no){

for(mm in 1:no) {

precBK[j,k,m,mm] <- prec[m,mm]*2*(k-1)/k # between-study precision matrix

}

}

}

}

#Consistency relations between basic parameters

#=====================================

for(i in 1:N2) {

tmp2[i] <- studyid1[i] # temp variable to identify study id, not used

for(k in 2:na[i]) {

md[s[i],k,out[i]] <- (d[out[i],t[i,k]] - d[out[i],t[i,1]])*equals(o[i],out[i]) + sw[s[i],k,out[i]]

w[s[i],k,out[i]] <- (delta[s[i],k,out[i]] - (d[out[i],t[i,k]] - d[out[i],t[i,1]]))*equals(o[i],out[i])

sw[s[i],k,out[i]] <- sum(w[s[i],1:k-1,out[i]])/(k-1)

}

}

#Constraints

# Effect in usual care arm is set to zero

# There are 9 interventions in total, but only 7 are trialled for each outcome, hence interventions 8 and 9 refer to the interventions were outcome information is not available.

d[1,1] <- 0

d[2,1] <- 0

d[3,1] <- 0

d[1,8] <- 0

d[1,9] <- 0

d[2,8] <- 0

d[2,9] <- 0

d[3,8] <- 0

d[3,9] <- 0

#Prior distributions and parameter to estimate

prec[1:no,1:no] <- inverse(sigma[,]) #hash out if using inverse-wishart (model 2a)

sd.se~ dunif(0, 2)

for(m in 1:no) {

prec.se[m] <- pow(sd.se,-2)

sigma[m,m] <- pow(sd[m],2) #hash out if using inverse-wishart (model 2a)

sd[m] ~ dunif(0, 2) #hash out if using inverse-wishart (model 2a)

for(j in 1:ns){

mu[j, m] ~ dnorm(0,0.001)

}

for(k in 2: nt.total[m]){

or[m,k] <- exp(d[m,k])

d[m,k] ~ dnorm(0,0.001)

}

}

#spherical parameterization (Wei and Higgins 2013)

#hash out if using inverse-wishart (model 2a)

pi <- 3.1415

for(i in 1:2) {

for(j in (i+1):no) {

sigma[i,j] <- rho[i,j]*sd[i]*sd[j]

sigma[j,i] <- sigma[i,j]

g[j,i] <- 0

a[i,j] ~ dunif(0, pi)

rho[i,j] <- inprod(g[,i], g[,j])

}

}

g[1,1] <- 1

g[1,2] <- cos(a[1,2])

g[2,2] <- sin(a[1,2])

g[1,3] <- cos(a[1,3])

g[2,3] <- sin(a[1,3])*cos(a[2,3])

g[3,3] <- sin(a[1,3])*sin(a[2,3])

#Inverse-Wishart prior (model 2a) hash

#prec[1:no, 1:no] ~ dwish(R[1:no,1:no],no)

#sigma[1:no,1:no] <- inverse(prec[,])

#between-study standard deviations

#sd[1] <- sqrt(sigma[1,1])

#sd[2] <- sqrt(sigma[2,2])

#sd[3] <- sqrt(sigma[3,3])

#between-study correlations

#rho[1,2] <- sigma[1,2]/(sd[1]*sd[2])

#rho[1,3] <- sigma[1,3]/(sd[1]*sd[3])

#rho[2,3] <- sigma[2,3]/(sd[2]*sd[3])

}

**Model 2: Data file 1 of 3**

list(

N1=45, #no of datapoints

N2=66, # no of studies x no of outcomes (22x3=66)

ns=22, # no of studies

no=3, #no of outcomes

nt.total =c(7,7,7), # no of interventions for outcomes 1, 2 and 3

mn.rhoW =c(0.184,-0.052,0.051), #mean of within-study correlations from IPD

se.rhoW = c(0.118,0.064,0.059,1), #se of within-study correlations from IPD

NA = c(2,2,2,2,2, 2,2,2,2,3, 2,2,2,2,2, 2,2,2,2,2, 2,2), # no. of arms in each study

#R = structure(.Data = c(1,0,0, 0,1,0, 0,0,1),.Dim = c(3,3)) # needed for model 2a

)

),

**Model 2: Data file 2 of 3**

| studyid[] | study[] | arm[] | y[,1] | y[,2] | y[,3] | se[,1] | se[,2] | se[,3] |
| --- | --- | --- | --- | --- | --- | --- | --- | --- |
| 9007 | 1 | 1 | 0.649184 | 0.893818 | NA | 0.127948 | 0.279791 | NA |
| 9007 | 1 | 2 | 1.125568 | 1.270463 | NA | 0.147352 | 0.28292 | NA |
| 26 | 2 | 1 | 2.627081 | 0.996333 | 0.67634 | 0.422747 | 0.238854 | 0.224238 |
| 26 | 2 | 2 | 2.577688 | 1.245216 | 1.052092 | 0.423468 | 0.260352 | 0.247644 |
| 9019 | 3 | 1 | 3.583519 | 1.43848 | 1.714798 | 0.71686 | 0.297284 | 0.362093 |
| 9019 | 3 | 2 | 3.555348 | 1.466337 | 1.132514 | 0.507093 | 0.213504 | 0.213527 |
| 48 | 4 | 1 | 4.691348 | 1.363305 | NA | 1.420686 | 0.337883 | NA |
| 48 | 4 | 2 | 4.70953 | 2.100061 | NA | 1.42057 | 0.432522 | NA |
| 344 | 5 | 1 | -2.584 | -2.89037 | NA | 0.518525 | 0.593171 | NA |
| 344 | 5 | 2 | -2.19722 | -2.21557 | NA | 0.430332 | 0.42994 | NA |
| 203 | 6 | 1 | NA | NA | -0.69315 | NA | NA | 0.181449 |
| 203 | 6 | 2 | NA | NA | 1.520952 | NA | NA | 0.222198 |
| 4 | 7 | 1 | 1.58045 | 0.395313 | NA | 0.293487 | 0.225192 | NA |
| 4 | 7 | 2 | 2.983154 | 0.899484 | NA | 0.512502 | 0.242107 | NA |
| 41 | 8 | 1 | NA | NA | -1.3689 | NA | NA | 0.207978 |
| 41 | 8 | 2 | NA | NA | -0.42652 | NA | NA | 0.187525 |
| 42 | 9 | 1 | NA | -0.41651 | -0.44425 | NA | 0.1663 | 0.166789 |
| 42 | 9 | 2 | NA | 0.377762 | 1.265666 | NA | 0.166221 | 0.197104 |
| 9002 | 10 | 1 | 4.297286 | NA | NA | 0.711901 | NA | NA |
| 9002 | 10 | 2 | 4.448516 | NA | NA | 0.71123 | NA | NA |
| 9002 | 10 | 3 | 3.976562 | NA | NA | 0.582738 | NA | NA |
| 279 | 11 | 1 | NA | -0.61904 | -1.38629 | NA | 0.331497 | 0.395285 |
| 279 | 11 | 2 | NA | 2.140066 | 2.140066 | NA | 0.528594 | 0.528594 |
| 9023 | 12 | 1 | 2.02004 | 1.440219 | NA | 0.34861 | 0.333812 | NA |
| 9023 | 12 | 2 | 2.524986 | 1.927793 | NA | 0.426773 | 0.393438 | NA |
| 49 | 13 | 1 | NA | 1.846879 | NA | NA | 0.152166 | NA |
| 49 | 13 | 2 | NA | 2.060979 | NA | NA | 0.165819 | NA |
| 345 | 14 | 1 | 2.519162 | -0.04485 | NA | 0.140164 | 0.077344 | NA |
| 345 | 14 | 2 | 2.656055 | 0.124258 | NA | 0.146303 | 0.07612 | NA |
| 28 | 15 | 1 | NA | NA | 0.716309 | NA | NA | 0.212838 |
| 28 | 15 | 2 | NA | NA | 0.977271 | NA | NA | 0.22683 |
| 35 | 16 | 1 | -0.59276 | NA | NA | 0.13252 | NA | NA |
| 35 | 16 | 2 | 0.064039 | NA | NA | 0.126971 | NA | NA |
| 9042 | 17 | 1 | NA | NA | -2.03143 | NA | NA | 0.265889 |
| 9042 | 17 | 2 | NA | NA | 0.043172 | NA | NA | 0.169677 |
| 10001 | 18 | 1 | NA | NA | 1.921813 | NA | NA | 0.309077 |
| 10001 | 18 | 2 | NA | NA | 3.100092 | NA | NA | 0.323272 |
| 12 | 19 | 1 | -0.85745 | -0.12783 | 0.300105 | 0.318954 | 0.292326 | 0.29502 |
| 12 | 19 | 2 | -0.45676 | 0.81831 | 0.916291 | 0.293198 | 0.309965 | 0.316228 |
| 29 | 20 | 1 | 2.174752 | 1.268511 | NA | 0.47194 | 0.377308 | NA |
| 29 | 20 | 2 | 3.068053 | 1.609438 | NA | 0.723364 | 0.387298 | NA |
| 24 | 21 | 1 | NA | 0.226982 | NA | NA | 0.092947 | NA |
| 24 | 21 | 2 | NA | 0.267138 | NA | NA | 0.091911 | NA |
| 14 | 22 | 1 | -1.27841 | -4.20469 | NA | 0.241066 | 0.822567 | NA |
| 14 | 22 | 2 | -1.43508 | -5.34233 | NA | 0.248807 | 1.417593 | NA |
| END |  |  |  |  |  |  |  |  |

**Model 2: Data file 3 of 3**

| studyid1[] | s[] | t[,1] | t[,2] | t[,3] | o[] | out[] | na[] |
| --- | --- | --- | --- | --- | --- | --- | --- |
| 9007 | 1 | 1 | 2 | NA | 1 | 1 | 2 |
| 9007 | 1 | 1 | 2 | NA | 2 | 2 | 2 |
| 9007 | 1 | 1 | 2 | NA | 0 | 3 | 2 |
| 26 | 2 | 1 | 2 | NA | 1 | 1 | 2 |
| 26 | 2 | 1 | 2 | NA | 2 | 2 | 2 |
| 26 | 2 | 1 | 2 | NA | 3 | 3 | 2 |
| 9019 | 3 | 1 | 2 | NA | 1 | 1 | 2 |
| 9019 | 3 | 1 | 2 | NA | 2 | 2 | 2 |
| 9019 | 3 | 1 | 2 | NA | 3 | 3 | 2 |
| 48 | 4 | 1 | 2 | NA | 1 | 1 | 2 |
| 48 | 4 | 1 | 2 | NA | 2 | 2 | 2 |
| 48 | 4 | 1 | 2 | NA | 0 | 3 | 2 |
| 344 | 5 | 1 | 2 | NA | 1 | 1 | 2 |
| 344 | 5 | 1 | 2 | NA | 2 | 2 | 2 |
| 344 | 5 | 1 | 2 | NA | 0 | 3 | 2 |
| 203 | 6 | 1 | 2 | NA | 0 | 1 | 2 |
| 203 | 6 | 1 | 2 | NA | 0 | 2 | 2 |
| 203 | 6 | 1 | 2 | NA | 3 | 3 | 2 |
| 4 | 7 | 1 | 3 | NA | 1 | 1 | 2 |
| 4 | 7 | 1 | 3 | NA | 2 | 2 | 2 |
| 4 | 7 | 1 | 3 | NA | 0 | 3 | 2 |
| 41 | 8 | 1 | 3 | NA | 0 | 1 | 2 |
| 41 | 8 | 1 | 3 | NA | 0 | 2 | 2 |
| 41 | 8 | 1 | 3 | NA | 3 | 3 | 2 |
| 42 | 9 | 1 | 3 | NA | 0 | 1 | 2 |
| 42 | 9 | 1 | 3 | NA | 2 | 2 | 2 |
| 42 | 9 | 1 | 3 | NA | 3 | 3 | 2 |
| 9002 | 10 | 1 | 3 | 4 | 1 | 1 | 3 |
| 9002 | 10 | 1 | 3 | 4 | 0 | 2 | 3 |
| 9002 | 10 | 1 | 3 | 4 | 0 | 3 | 3 |
| 279 | 11 | 1 | 4 | NA | 0 | 1 | 2 |
| 279 | 11 | 1 | 4 | NA | 2 | 2 | 2 |
| 279 | 11 | 1 | 4 | NA | 3 | 3 | 2 |
| 9023 | 12 | 1 | 4 | NA | 1 | 1 | 2 |
| 9023 | 12 | 1 | 4 | NA | 2 | 2 | 2 |
| 9023 | 12 | 1 | 4 | NA | 0 | 3 | 2 |
| 49 | 13 | 1 | 4 | NA | 0 | 1 | 2 |
| 49 | 13 | 1 | 4 | NA | 2 | 2 | 2 |
| 49 | 13 | 1 | 4 | NA | 0 | 3 | 2 |
| 345 | 14 | 1 | 5 | NA | 1 | 1 | 2 |
| 345 | 14 | 1 | 5 | NA | 2 | 2 | 2 |
| 345 | 14 | 1 | **8** | NA | 0 | 3 | 2 |
| 28 | 15 | 1 | **8** | NA | 0 | 1 | 2 |
| 28 | 15 | 1 | **8** | NA | 0 | 2 | 2 |
| 28 | 15 | 1 | 5 | NA | 3 | 3 | 2 |
| 35 | 16 | 1 | 6 | NA | 1 | 1 | 2 |
| 35 | 16 | 1 | 6 | NA | 0 | 2 | 2 |
| 35 | 16 | 1 | 6 | NA | 0 | 3 | 2 |
| 9042 | 17 | 1 | 6 | NA | 0 | 1 | 2 |
| 9042 | 17 | 1 | 6 | NA | 0 | 2 | 2 |
| 9042 | 17 | 1 | 6 | NA | 3 | 3 | 2 |
| 10001 | 18 | 1 | **9** | NA | 0 | 1 | 2 |
| 10001 | 18 | 1 | **9** | NA | 0 | 2 | 2 |
| 10001 | 18 | 1 | 7 | NA | 3 | 3 | 2 |
| 12 | 19 | 2 | 3 | NA | 1 | 1 | 2 |
| 12 | 19 | 2 | 3 | NA | 2 | 2 | 2 |
| 12 | 19 | 2 | 3 | NA | 3 | 3 | 2 |
| 29 | 20 | 2 | 5 | NA | 1 | 1 | 2 |
| 29 | 20 | 2 | 5 | NA | 2 | 2 | 2 |
| 29 | 20 | 2 | **8** | NA | 0 | 3 | 2 |
| 24 | 21 | 4 | 6 | NA | 0 | 1 | 2 |
| 24 | 21 | 4 | 6 | NA | 2 | 2 | 2 |
| 24 | 21 | 4 | 6 | NA | 0 | 3 | 2 |
| 14 | 22 | 3 | 7 | NA | 1 | 1 | 2 |
| 14 | 22 | 3 | 7 | NA | 2 | 2 | 2 |
| 14 | 22 | 3 | **9** | NA | 0 | 3 | 2 |
| END |  |  |  |  |  |  |  |

**Appendix 2: WinBUGS code for model 3 code**

**#Model 3 code**

**Model { #model 3**

**#Likelihood for arm level data**

**#=======================**

for(i in 1:N1){

tmp1[i] <- studyid[i] # study id not used in the model

y[i,1:3] ~ dmnorm(mean.y[study[i],arm[i],1:3],omega[i,,]) # multivariate likelihood

omega[i,1:3,1:3] <- inverse(cov.mat[i,,]) # within-study precision matrix

#define elements of within-study covariance matrix

cov.mat[i,1,1] <- pow(se[i,1],2)

cov.mat[i,2,2] <- pow(se[i,2],2)

cov.mat[i,3,3] <- pow(se[i,3],2)

cov.mat[i,1,2] <- se[i,1]*se[i,2]*cor[i,1]

cov.mat[i,1,3] <- se[i,1]*se[i,3]*cor[i,2]

cov.mat[i,2,3] <- se[i,2]*se[i,3]*cor[i,3]

cov.mat[i,2,1] <- cov.mat[i,1,2]

cov.mat[i,3,1] <- cov.mat[i,1,3]

cov.mat[i,3,2] <- cov.mat[i,2,3]

for(m in 1:no){

se[i,m] ~ dnorm(0, prec.se[m])I(0,) # input missing standard errors

unif.a[i,m] <- mn.rhoW[m] - (sqrt(12)*se.rhoW[m]/2) # parameter a of uniform distribution

unif.b[i,m] <- mn.rhoW[m] +(sqrt(12)*se.rhoW[m]/2) # parameter b of uniform distribution

cor[i,m] ~ dunif(unif.a[i,m], unif.b[i,m]) # within-study correlation model

}

}

for(j in 1:ns){

for(k in 1:NA[j]) **{**

for(m in 1:no){

mean.y[j,k,m] <- mu[j,m] + delta[j,k,m] # define study-specific treatment effects

}

}

**}**

**#Random effects between-study model**

**#================================**

for(j in 1:ns) {

tmp2[j] <- studyid1[j]

tmp3[j] <- s[j]

for(m in 1:no) **{**

delta[j,1,m] <-0 #delta's in control arm to zero for all outcomes

w[j,1,m] <-0 #multi-arm adjustment in control group set to zero

**}**

for(k in 2:na2[j]) **{**

delta[j,k,1:no] ~ dmnorm(md[j,k,1:no],precBK[j,k,1:no,1:no]) #trial specific trt effects drawn from mvn distribution

for(m in 1:no)**{**

md[j,k,m] <- (d[m,t[j,k]] - d[m,t[j,1]])+ sw[j,k,m] #consistency equations

w[j,k,m] <- delta[j,k,m] - (d[m,t[j,k]] - d[m,t[j,1]]) #multi-arm adjustemnt for treatment k

sw[j,k,m] <- sum(w[j,1:k-1,m])/(k-1)

for(mm in 1:no) {

precBK[j,k,m,mm] <- prec[m,mm]*2*(k-1)/k

}

}

**}**

**}**

#Constraints

#There are 8 trts in total, but only 7 treatments are trialled for each outcome, hence 8 is code

# when trt has not been considered for the outcome. also effect in usual care arm is set to zero

d[1,1] <- 0

d[2,1] <- 0

d[3,1] <- 0

#Prior distributions and parameter to estimate

prec[1:no,1:no] <- inverse(sigma[,]) #hash out if using inverse-wishart (model 2a)

sd.se~ dunif(0, 2)

for(m in 1:no) {

prec.se[m] <- pow(sd.se,-2)

sigma[m,m] <- pow(sd[m],2) #hash out if using inverse-Wishart (model 2a)

sd[m] ~ dunif(0, 2) #hash out if using inverse-Wishart (model 2a)

for(j in 1:ns){

mu[j, m] ~ dnorm(0,0.0001)

}

}

#spherical parameterization (Wei and Higgins 2013)

pi <- 3.1415

for(i in 1:2) {

for(j in (i+1):no) {

sigma[i,j] <- rho[i,j]*sd[i]*sd[j]

sigma[j,i] <- sigma[i,j]

g[j,i] <- 0

a[i,j] ~ dunif(0, pi)

rho[i,j] <- inprod(g[,i], g[,j])

}

}

g[1,1] <- 1

g[1,2] <- cos(a[1,2])

g[2,2] <- sin(a[1,2])

g[1,3] <- cos(a[1,3])

g[2,3] <- sin(a[1,3])*cos(a[2,3])

g[3,3] <- sin(a[1,3])*sin(a[2,3])

**# Borrowing information across outcomes**

**#===========================================**

#intervention effects exponentiated and prior distributions

for(k in 2: nt){

for(m in 1:no) {

meanD[m,k-1] <- alpha[k-1] + gamma[m] #outcome and intervention effects

d[m,k] ~ dnorm(meanD[m,k-1], prec.btw) #trt effects

OR[m,k-1] <- exp(meanD[m,k-1]) # extrapolated effects of interest in model 3

or[m,k] <- exp(d[m,k]) #shrunken estimates based on equation (7)

}

}

for(m in 1:no) {gamma[m] ~ dnorm(0, 0.0001) }

for(k in 1:(nt-1)) {alpha[k] ~ dnorm(0, 0.0001) }

prec.btw <- pow(sd.btw,-2)

sd.btw ~ dunif(0, 2)

**}**

#END

**Model 3: Data file 1 of 3**

list(

N=45, #no of datapoints

ns=22, # no of studies

no=3, #no of outcomes

nt =9,

mn.rhoW =c(0.184,-0.052,0.051), #mean of within-study correlations from IPD

se.rhoW = c(0.118,0.064,0.059,1), #se of within-study correlations from IPD

NA = c(2,2,2,2,2, 2,2,2,2,3, 2,2,2,2,2, 2,2,2,2,2, 2,2), # no. of arms in each study

)

**Model 3: Data file 2 of 3 is the same as in model 2**

**Model 3: Data file 3 of 3**

| studyid1[] | s[] | t[,1] | t[,2] | t[,3] | na2[] |
| --- | --- | --- | --- | --- | --- |
| 9007 | 1 | 1 | 2 | NA | 2 |
| 26 | 2 | 1 | 2 | NA | 2 |
| 9019 | 3 | 1 | 2 | NA | 2 |
| 48 | 4 | 1 | 2 | NA | 2 |
| 344 | 5 | 1 | 2 | NA | 2 |
| 203 | 6 | 1 | 2 | NA | 2 |
| 4 | 7 | 1 | 3 | NA | 2 |
| 41 | 8 | 1 | 3 | NA | 2 |
| 42 | 9 | 1 | 3 | NA | 2 |
| 9002 | 10 | 1 | 3 | 4 | 3 |
| 279 | 11 | 1 | 4 | NA | 2 |
| 9023 | 12 | 1 | 4 | NA | 2 |
| 49 | 13 | 1 | 4 | NA | 2 |
| 345 | 14 | 1 | 5 | NA | 2 |
| 28 | 15 | 1 | 6 | NA | 2 |
| 35 | 16 | 1 | 7 | NA | 2 |
| 9042 | 17 | 1 | 7 | NA | 2 |
| 10001 | 18 | 1 | 8 | NA | 2 |
| 12 | 19 | 2 | 3 | NA | 2 |
| 29 | 20 | 2 | 5 | NA | 2 |
| 24 | 21 | 4 | 7 | NA | 2 |
| 14 | 22 | 3 | 9 | NA | 2 |
| END |  |  |  |  |  |
